# A Nrf-2 Stimulatory Hydroxylated Cannabidiol Derivative from Hemp (*Cannabis sativa*)

**DOI:** 10.1021/acs.jnatprod.1c01198

**Published:** 2022-03-22

**Authors:** Giuseppina Chianese, Carmina Sirignano, Emanuele Benetti, Vittoria Marzaroli, Juan A. Collado, Lauren de la Vega, Giovanni Appendino, Eduardo Muñoz, Orazio Taglialatela-Scafati

**Affiliations:** † Department of Pharmacy, School of Medicine and Surgery, University of Naples Federico II, Via D. Montesano 49, 80131 Napoli, Italy; ‡ Indena SpA, Via Don Minzoni, 6, 20049 Settala, Milan, Italy; # Instituto Maimónides de Investigación Biomédica de Córdoba (IMIBIC), Avenida Menéndez Pidal, s/n, 14004 Córdoba, Spain; § Departamento de Biología Celular, Fisiología e Inmunología, Universidad de Córdoba, Spain, and Hospital Universitario Reina Sofía, 14014 Córdoba, Spain; ∧ Jacqui Wood Cancer Centre, Division of Cellular Medicine, School of Medicine, University of Dundee, James Arnott Drive, Ninewells Hospital, DD2 1UB Dundee, U.K.; ∥ Dipartimento di Scienze del Farmaco, Università del Piemonte Orientale, Largo Donegani 2, 28100, Novara, Italy

## Abstract

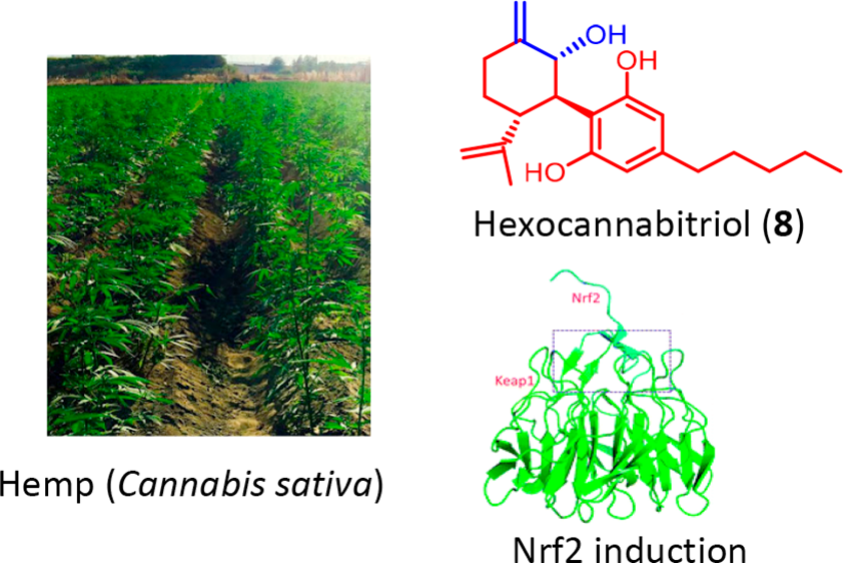

A phytochemical analysis of mother liquors obtained from crystallization of CBD from hemp (*Cannabis sativa*), guided by LC-MS/MS and molecular networking profiling and completed by isolation and NMR-based characterization of constituents, resulted in the identification of 13 phytocannabinoids. Among them, anhydrocannabimovone (**5**), isolated for the first time as a natural product, and three new hydroxylated CBD analogues (1,2-dihydroxycannabidiol, **6**, 3,4-dehydro-1,2-dihydroxycannabidiol, **7**, and hexocannabitriol, **8**) were obtained. Hexocannabitriol (**8**) potently modulated, in a ROS-independent way, the Nrf2 pathway, outperforming all other cannabinoids obtained in this study and qualifying as a potential new chemopreventive chemotype against cancer and other degenerative diseases.

Cannabis (*Cannabis sativa* L., Cannabaceae) continues to be a socially divisive plant because of the illegal commerce and consumption of marijuana, its narcotic chemotype, as a recreational drug. Legal and social concerns aside, the past two decades have witnessed a renaissance of medicinal interest in *C. sativa*, triggered by a growing evidence of its clinical efficacy in different pathological conditions (e.g., chemotherapy-induced nausea and vomiting, chronic pain, and spasticity associated with multiple sclerosis).^1^ Interaction with cannabinoid (CB) receptors is mainly responsible for these activities, an observation that highlights the critical role played by Δ^9^-THC (**1**, Figure 1), the archetypal phytocannabinoid that binds with high affinity to the ligand-recognizing site of CB_1_.^2^ On the other hand, there is also growing evidence that the pharmacological/biomedical potential of *C. sativa* extends substantially beyond the biological profile of Δ^9^-THC (**1**, Figure 1) and its interactions with CB receptors. Thus, despite the contrasting pro- and anticonvulsivant properties of Δ^9^-THC,^3^ cannabidiol (CBD, **2**) has shown clinical efficacy in the management of genetic forms of juvenile epilepsy (Lennox-Gastaut and Dravet syndromes),^4^ and its pharmacological profile does not overlap with that of Δ^9^-THC in terms of interaction with GPCR (CBs, 5-HT receptors), ion channels (thermo-TRPs), or transcription factors (PPARs).^4^

**Figure 1:**
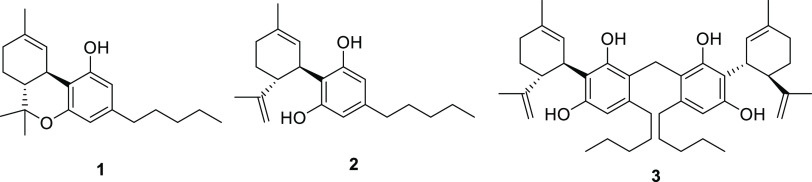
Structures of Δ^9^-THC (**1**), CBD (**2**), and cannabitwinol (**3**).

The diversity of cannabinoid targets has provided a strong rationale to explore the occurrence of minor and trace phytocannabinoids, with the aim of systematically disclosing the pharmacological parameters of compounds with even minor structural changes. In line with this goal, in the past few years this has led to the discovery of chemotypes missed in the classic studies of the 1960s and 1970s. Included are ester derivatives with monoterpenoids of acidic phytocannabinoids^5^ and analogues with shortened (3C and 4C)^6^ or elongated (6C,^7^ 7C^8^) linear alkyl side chains or with oxidatively rearranged terpenoid moieties.^9,10^ As a result, the inventory of structurally characterized phytocannabinoids has grown to over 150 members,^11^ and significant clues for a better definition of their structure–activity relationships have been obtained.

In the framework of the phytochemical analysis of hemp, our group has recently reported the characterization of cannabitwinol (CBDD, **3**),^12^ a dimeric phytocannabinoid characterized by two CBD units connected by a methylene bridge. This structural duplication is associated with a substantial change of the modulation of thermo-TRPs with an interesting selectivity for channels activated by a decrease (TRPM8 and TRPA1) rather than by an increase of temperature (TRPV1–V4).^12^ Spurred by the structural and biological novelty of CBDD, it has been possible to capitalize on LC-MS/MS and molecular networking profiling to identify additional trace cannabinoids from the mother liquors obtained from CBD crystallization from hemp extracts. Isolation and NMR-based characterization of the extract constituents resulted in the discovery, along with known compounds, of three new CBD analogues (**6**–**8**), one of which (**8**) showed very promising Nrf2 activation properties.

## Results and Discussion

**Figure 2:**
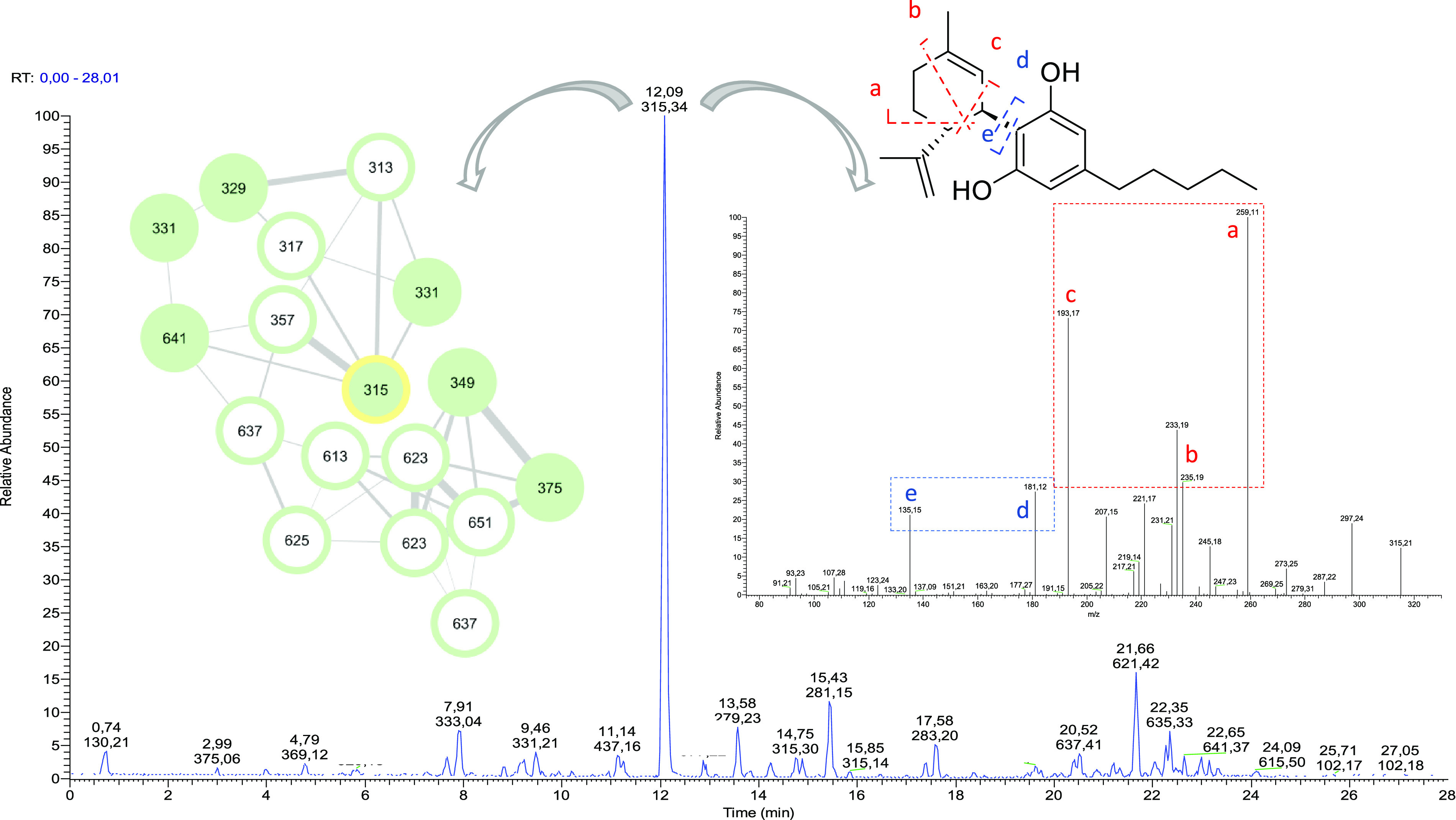
LC-MS chromatogram of the *C. sativa* mother liquors extract and (left) selected cannabinoid cluster from MS/MS-based molecular network (nodes are labeled with the parent *m*/*z* ratio; edge thickness is related to the cosine similarity score; colors: green for compounds isolated by RP-HPLC and fully assigned by HR-MSMS and NMR; white for the unassigned nodes; yellow ring for cannabidiol); (right) LC-MS/MS spectrum of cannabidiol (**2**).

A cannabinoid-rich extract was obtained from hemp threshing residues, used as sources of CBD (**2**), extracted in a percolator with 80% ethanol at ambient temperature. The extract was diluted with aqueous ethanol (70%), decarboxylated, and partitioned with *n*-hexane. The polar organic phases were concentrated and then chromatographed on silica gel. The phytocannabinoid-rich fractions were concentrated to obtain a soft residue and dried to obtain a powder. Cannabidiol (**2**) was crystallized from heptane, and the mother liquors were analyzed via HPLC-mass spectrometry in the positive scan mode. As expected, the main component of the mixture was CBD (**2**), *m*/*z* 315 [M + H]^+^, for which the MS/MS fragmentation pattern (Figure 2), in agreement with literature data,^13^ included fragments corresponding to the resorcinol core (*m*/*z* 181 [M + H]^+^) and the *p*-menthane moiety (*m*/*z* 135 [M + H]^+^), as well as fragments derived from partial cleavage of the terpene moiety (*m*/*z* 259, 235, and 193 ([M + H]^+^) from the parent ion (Figure 2).^13^

The fraction was dereplicated by adopting a combined LC-MS/MS and molecular networking (MS^2^-MN) approach.^14^ Molecular networking (MN) is a computational strategy that facilitates visualization for untargeted mass spectrometric analysis using the Global Natural Product Social Molecular Network (GNPS), a computational algorithm that compares the degree of similarity of MS/MS spectra with those deposited in the data set, allowing users to annotate and identify known metabolites as well as structural analogues.^15^

The full network obtained from the sample analyzed included a large cluster containing mainly phytocannabinoid derivatives (Figure 2). This preliminary information guided an accurate manual analysis of the HRMSMS that resulted in the putative identification of several compounds, as reported in Table 1. Some nodes in the phytocannabinoid network could be associated with molecular weights and fragmentation patterns attributable to known phytocannabinoids (Table 1); however their annotation needed further investigation, suggesting the presence of unknown analogues. Thus, the nodes *m*/*z* 349.2 (*t*_R_ 3.96) and *m/z* 331.2 (*t*_R_ 7.24) showed mass values and fragmentation patterns superimposable on those of the THC derivatives cannabiripsol (CBR) and 10α-hydroxy-Δ^9,11^-hexahydrocannabinol,^16^ respectively, but the origin of the extract from a non-narcotic biomass made this identification unlikely, rather suggesting the presence of new hydroxylated CBD derivatives.

**Table 1:** Identified Components of the Hemp Extract Analyzed via LC-MS/MS and Molecular Networking (MN) and the Main Parameters Supporting Their Identification^a^,^b^

family	assignment	formula	*t*_R_ (min)	precursor ion (*m*/*z*)	fragments (*m*/*z*)	identification criteria
cannabinoid	cannabielsoic acid (**4b**)	C_22_H_30_O_5_	2.99	375.06	357.14, 339.14	MN - isolation
cannabinoid	new cannabinoid (**6**)	C_21_H_32_O_4_	3.96	349.21	331.20, 313.22, 273.28, 231.25, 193.24, 181.19	MN - isolation
flavonoid	cannflavin B	C_21_H_20_O_6_	4.79	369.12	313.07, 217.22, 133.0	standard - isolation
cannabinoid	new cannabinoid (**7**)	C_21_H_30_O_4_	5.83	347.22	329.20, 311.22	isolation
cannabinoid	new cannabinoid (**8**)	C_21_H_30_O_3_	7.24	331.22	313.13, 193.12	MN - isolation
cannabinoid	*not assigned*		7.91	333.04	315.29, 277.34, 251.12, 193.24	
cannabinoid	cannabielsoin (**4a**)	C_21_H_30_O_3_	9.46	331.21	313.20, 271.15, 205.14, 181.14	MN - isolation
cannabinoid	anhydrocannabimovone (**5**)	C_21_H_28_O_3_	9.92	329.20	311.23, 287.16, 193.19, 181.17	MN - isolation
flavonoid	cannflavin A	C_26_H_28_O_6_	11.14	437.16	381.11, 327.18, 313.16	standard - isolation
cannabinoid	cannabidiolic acid	C_22_H_30_O	11.24	359.03	341.38, 219.13, 193.22	standard - isolation
cannabinoid	cannabidiol (**2**)	C_12_H_30_O_2_	12.09	315.34	297.24, 259.10, 235.20, 193.17, 181.12, 135.15	standard - isolation
fatty acid	γ-linolenic acid	C_18_H_30_O_2_	13.58	279.23	261.19, 243.20, 223.17	standard
cannabinoid	cannabinodiol	C_21_H_26_O_2_	13.84	311.28	293.19, 283.17, 173.15	standard
cannabinoid	Δ^9^-tetrahydrocannabinol (**1**)	C_21_H_30_O_2_	14.75	315.30	259.11, 245.18, 235.19, 193.11, 181.12, 135.16	standard
cannabinoid	cannabidiphorol	C_23_H_34_O_2_	14.88	343.20	287.12, 263.17, 221.13, 209.10, 193.08, 135.20	tentative identification by LC-MS/MS
fatty acid	linoleic acid	C_18_H_32_O_2_	15.43	281.15	263.20, 245.18, 225.17	standard
cannabinoid	cannabichromene	C_21_H_30_O_2_	15.85	315.14	259.11, 245.18, 233.21, 193.11, 181.12, 135.16	standard
fatty acid	oleic acid	C_18_H_34_O_2_	17.58	283.20	265.23, 247.24	standard
cannabinoid	cannabitwinol (**3**)	C_43_H_60_O_4_	22.65	641.37	598.39, 327.13, 315.26	MN - standard

a Compounds are listed in order of LC-MS elution. All mass peaks are [M + H]^+^ adducts.

b MS/MS spectra are reported in the Supporting Material.

**Figure 3:**
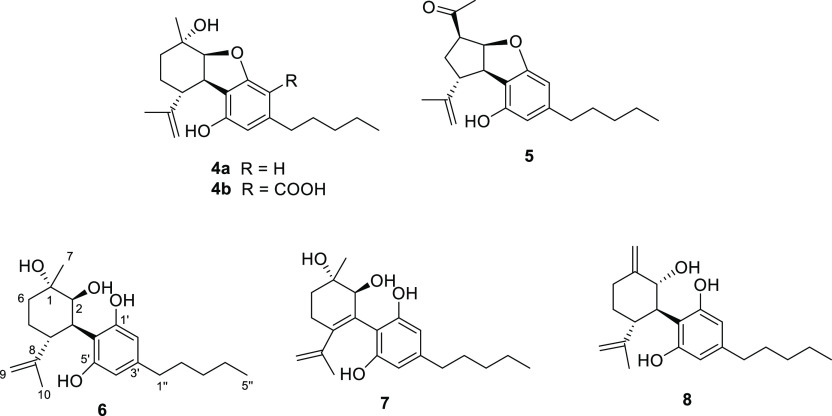
Structures of selected phytocannabinoids obtained as constituents of the mother liquors obtained from the crystallization of CBD (**2**).

As reported in Table 1, using standards available from previous studies, several assignments made could be confirmed, but other compounds could only be identified after isolation and detailed NMR spectroscopic investigation. Toward this end, the mother liquors from the crystallization of CBD (**2**) were subjected to MPLC-DAD on a C_18_ column followed by repeated HPLC purifications, guided by the preliminary LC profile. In this way, several compounds were obtained. In addition to three unsaturated fatty acids and the prenylated flavones cannflavins A and B,^17^ the metabolomic profile of mother liquors also included 12 phytocannabinoids (Figure 3) in addition to CBD (**2**), namely, the corresponding acid (CBDA), the heptyl homologue cannabidiphorol (CBDP),^8^ cannabielsoin (CBE, **4a**) and its corresponding acid (**4b**, CBEA),^18^ cannabichromene (CBC), cannabinodiol (CBND), Δ^9^-tetrahydrocannabinol^19^ (**1**, Δ^9^-THC, present in trace amounts, as expected), and the dimeric cannabitwinol (**3**, CBDD).^12^ In addition, also identified were, from MN and subsequent isolation, anydrocannabimovone (**5**),^9,20^ a tricyclic CBD analogue previously reported as a CB_1_/CB_2_ low micromolar agonist obtained as byproduct during attempts to prepare cannabimovone from CBD.^9^ This is the first report of **5** as a natural product.

The structures of these known compounds were confirmed on the basis of the comparison of their chromatographic (LC-HRMS), spectroscopic (MS/MS and NMR), and optical rotation data with the published values (Table 1). The chromatographic purifications afforded also, in the pure form, the three phytocannabinoids remaining unassigned in the preliminary LC-MS/MS analysis (see Table 1). Their structures were elucidated as 1,2-dihydroxycannabidiol (**6**), 3,4-dehydro-1,2-dihydroxycannabidiol (**7**), and the compound 10α-hydroxy-Δ^1,7^-hexahydrocannabinodiol, for which the trivial name hexocannabitriol (**8**) is proposed (Figure 3).

**Figure 4:**
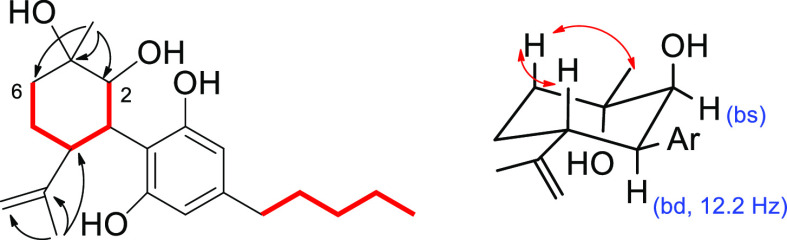
Diagnostic 2D NMR correlations detected for **6**. (Left) COSY (red bolded) and key HMBC (arrows) correlations. (Right) Key NOESY (red arrows) correlations.

1,2-Dihydroxycannabidiol (**6**) was isolated as a pale yellow amorphous solid with the molecular formula C_21_H_32_O_4_ (HRESIMS). The ^1^H NMR spectrum of **6** showed typical features of a CBD derivative, including two signals for a resorcinol unit (δ_H_ 6.12 and 6.29) and the signals of *n*-pentyl and of the monoterpenyl units. However, resonances of this moiety showed significant differences compared to CBD, since they lacked the sp^2^ methine signal, replaced by a sp^3^ oxymethine (δ_H_ 3.77), and showed an upfield shift of one of the two methyl singlets. The COSY spectrum was instrumental in building a spin system spanning from the oxymethine H-2 to H_2_-6 (Figure 4). After association of the proton signals to the directly attached carbon atoms via the HSQC spectrum (Tables 2 and 3), the planar structure of **6** could be deduced by using the HMBC spectrum. Thus, the ^2,3^*J*_C,H_ correlations of H_3_-7 with C-2, C-6, and the oxygenated and nonprotonated C-1 and those of H_3_-10 with C-9, C-8, and C-6 (Figure 4) were diagnostic of a dioxygenated menthyl architecture for the terpenoid moiety of **6**.

**Table 2:** ^1^H (700 MHz) NMR Data of Compounds **6**–**8** in CDCl_3_

	**6**	**7**	**8**
position	δ_H_, mult., *J* in Hz	δ_H_, mult., *J* in Hz	δ_H_, mult., *J* in Hz
1			
2	3.77, bs	3.78, s	4.63, bd, 10.6
3	3.96, bd, 12.2		3.08, t, 10.6
4	3.10, ddd, 12.5, 12.2, 3.5		3.31, ddd, 11.0, 10.6, 3.5
5a	1.61^a^	2.33^a^	1.45^a^
5b	1.83^a^	2.66^a^	1.78^a^
6a	1.62^a^	1.75^a^	2.35^a^
6b	1.97^a^	2.06^a^	
7a	1.30, s	1.37, s	4.85, s
7b			5.08, s
8			
9a	4.49, bs	4.76, bs	4.49, bs
9b	4.75, bs	4.78, bs	4.65, bs
10	1.55, s	1.66, s	1.56, s
1′			
2′	6.12, s	6.30, s	6.09, s
3′			
4′	6.29, s	6.31, s	6.20, s
5′			
6′			
1″	2.43, t, 7.2	2.48, t, 7.2	2.41, m
2″	1.57^a^	1.60^a^	1.56^a^
3″	1.31^a^	1.31^a^	1.33^a^
4″	1.33^a^	1.34^a^	1.31^a^
5″	0.87, t, 7.0	0.88, t, 7.0	0.89, t, 7.0

a Overlapped.

**Table 3:** ^13^C (175 MHz) NMR Data of Compounds **6**–**8** in CDCl_3_

	**6**	**7**	**8**
position	δ_C_, type	δ_C_, type	δ_C_, type
1	71.7, C	70.9, C	150.3, C
2	80.1, CH	76.5, CH	73.0, CH
3	36.8, CH	121.9, C	48.0, CH
4	40.2, CH	144.7, C	46.6, CH
5	27.2, CH_2_	27.5, CH_2_	34.0, CH_2_
6	33.0, CH_2_	29.2, CH_2_	33.9, CH_2_
7	27.5, CH_3_	26.1, CH_3_	104.7, CH_2_
8	147.8, C	148.9, C	147.8, C
9	110.8, CH_2_	113.5, CH_2_	110.4, CH_2_
10	18.2, CH_3_	21.3, CH_3_	19.05, CH_3_
1′	153.0, C	153.9, C	155.0, C
2′	107.4, CH	107.8, CH	109.0, CH
3′	143.2, C	144.9, C	143.0, C
4′	110.9, CH	107.1, CH	108.7, CH
5′	157.6, C	153.0, C	154.7, C
6′	112.5, C	113.4, C	110.7, C
1″	35.2, CH_2_	35.9, CH_2_	35.2, CH_2_
2″	27.4, CH_2_	27.4, CH_2_	27.4, CH_2_
3″	31.3, CH_2_	30.4, CH_2_	31.3, CH_2_
4″	22.3, CH_2_	22.3, CH_2_	22.3, CH_2_
5″	14.0, CH_3_	14.0, CH_3_	14.0, CH_3_

The structure of compound **6** includes four adjacent stereogenic carbons (C-1 to C-4), for which relative configuration was assigned on the basis of proton–proton coupling constant values and NOESY cross-peaks. The *trans*-diaxial orientation of the H-3/H-4 pair was inferred from *J*_H-3/H-4_ = 12.2 Hz (Figure 4); conversely, the very small coupling constant (ca. 0) of the H-2/H-3 pair indicated the *cis* equatorial-axial relationship of the corresponding protons. Finally, the NOESY cross-peaks of both H-4 and H_3_-7 with H-6ax indicated that these three protons have the same relative orientation. Due to the very high optical purity of natural CBD,^21^ the absolute configuration of **6** was assumed to be the same as that of CBD.

The dihydroxylation of the endocyclic double bond of CBD to generate **6** is similar to that generating cannabiripsol from Δ^9^-THC in high-potency marijuana.^16^ The dihydroxylation of the menthyl ring caused a complete decay of the characteristic Δ^9^-THC agonistic activity on CB_1_ and CB_2_ receptors, and cannabiripsol was almost completely inactive on these end points.^16^

3,4-Dehydro-1,2-dihydroxycannabidiol (**7**), the 3,4-unsaturated analogue of **6**, was also isolated in small amounts as a pale yellow amorphous solid. The molecular formula C_21_H_30_O_4_ (HRESIMS) showed one additional unsaturation degree, located at the C-3,C-4-positions following NMR analysis.

In particular, compared to that of **6**, the ^1^H NMR spectrum of **7** lacked the midfield resonances of H-3 and H-4 and showed a significant downfield shift of the H_2_-5 and H_2_-9 proton signals. The COSY and HSQC spectra allowed assignments of all protons and corresponding carbon atoms, while the HMBC spectra further confirmed the location of the tetrasubstituted double bond. Diagnostic HMBC cross-peaks were those of H_3_-10 with the three sp^2^ carbons C-4 (δ_C_ 144.7), C-8, and C-9 as well as those of H-2 with C-3 (δ_C_ 121.9), C-4, and C-6′. Finally, the NOESY cross-peaks H-2/H-6ax and H_3_-7/H-5ax were indicative of the *trans*-relationship between the two OH groups. Compound **7** is the CBD analogue of the THC oxidized derivative named cannabitriol,^22^ found both as a natural product and as a metabolite of Δ^9^-THC in marijuana consumers.^23^

Hexocannabitriol (**8**), C_21_H_30_O_3_ by HRESIMS, was isolated as an optically active pale yellow amorphous solid. Its ^1^H NMR spectrum (Table 2) showed typical signals of pentyl and resorcinyl moieties of phytocannabinoids. The signals associated with the terpenyl part included two sp^2^ methylenes, one oxymethine (δ_H_ 4.63), and a single methyl singlet resonating in the allylic region (δ_H_ 1.56). Using the COSY correlations, all the proton multiplets of this moiety could be organized within a single spin system, connecting the oxymethine proton (H-2) to H_2_-6. The 2D NMR HSQC spectrum confirmed the presence of an oxymethine (δ_C_ 73.0) and of two sp^2^ methylenes (δ_C_ 104.7 and 110.4) in the terpenoid moiety. The network of HMBC cross-peaks was instrumental in defining the *p*-menthyl architecture, which included a Δ^1,7^ hexomethylene (correlations of H_2_-7 with C-1, C-2, and C-6 and of H_3_-10 with C-4, C-8, and C-9). The large *J* values (10.6 Hz) of the H-2/H-3 and H-3/H-4 coupling constants suggested an axial orientation for these protons and, consequently, a *trans,trans* relative orientation. The absolute configuration of **8** was assumed to be the same as that of CBD.

Compounds **6** and **8** could derive mechanistically from the complementary opening of a putative 1,2-α-epoxide of CBD. Acidic opening would generate a C-1 cation and next be deprotonated to afford **8**. Conversely, opening via an S_N_2 mechanism with configurational inversion at C-2 could generate **6** by water attack, and cannabielsoin (**4a**) by intramolecular attack of the resorcinolic phenol group. A similar process could underlie the formation of the glycol system of **7**, featuring the Δ^3,4^-unsaturation typical of the early synthetic cannabinoids by Adams.^24^ Interestingly, analogues of **6**–**8** from the Δ^9^-THC series are all known, having been isolated from high-potency *C. sativa*.^25^ The aerobic oxidation of CBD involves the resorcinolic core rather than the terpenyl moiety, but epoxidation with peracids of dimethyl-CBD occurs selectively at the endocyclic double bond, with methylation of the phenolic hydroxy groups being necessary to avoid oxidation of the resorcinyl core as well as the intramolecular opening of the epoxide ring.^26^ Given their occurrence in trace amounts, the three new compounds (**6**–**8**) could, in principle, derive from the autoxidation of CBD. On the other hand, the failure to detect CBDQ in the mother liquors suggests a limited level of autoxidation in the cannabinoid fraction and therefore an enzymatic origin for these three new compounds.

While no specific high-affinity target of CBD has been identified so far, there is a growing interest for the reported antioxidant and anti-inflammatory properties of some non-psychotropic phytocannabinoids.^27^ CBD and its analogues can regulate the redox balance by interacting with components of the redox system directly or indirectly, through regulating the expression of antioxidant enzymes. Thus, CBD, like other phenolic antioxidants, interrupts free-radical chain reactions and reduces the production of reactive oxygen species (ROS) by chelating transition metal ions involved in the Fenton reaction.^28^ On the other hand, CBD also modulates the expression of antioxidant enzymes^29^ by regulating the levels of both transcription factors Nrf2 (nuclear erythroid 2-related factor) and BACH1 (BTB domain and CNC homologue 1), two master regulators of oxidative stress responses.^30,31^ Nrf2 is a key regulator of the cellular antioxidant response controlling the transcription of a panel of cytoprotective and antioxidant genes.^32^ Nrf2 is controlled mainly at the protein level, and its main regulator, KEAP1 (Kelch-like ECH-associated protein 1), is a substrate adaptor for the Cul3-based E3 ubiquitin ligase. In normal conditions, KEAP1 targets Nrf2 for proteasomal degradation, keeping its levels low. Cell exposure to ROS results in an impairment of Nrf2 degradation by KEAP1, leading to Nrf2 stabilization, its nuclear translocation, and activation of the pathway.

BACH1 acts as a negative regulator of the Nrf2 pathway by competing for the binding to the promoters of a subset of Nrf2 target genes such as HMOX1 and p62. The main BACH1 target gene is HMOX1, and, while Nrf2 activators induce the expression of a panel of cytoprotective genes, BACH1 inhibitors activate only a subset of these genes, although they are very potent at inducing HMOX1.^32^ Nrf2 activation and/or BACH1 inhibition provide cytoprotection against numerous chronic conditions characterized by inflammatory and pro-oxidant components.

CBD increases the activity of glutathione peroxidase and reductase and, in human cardiomyocytes, was found to increase the mRNA level of superoxide dismutase (SOD).^29^ This activity has been related to an activation of Nrf2,^30,31^ although Muñoz and co-workers demonstrated that in keratinocytes CBD is a weak Nrf2 activator but a good BACH1 inhibitor, selectively stimulating the expression of a limited subset of Nrf2-induced target genes such as HMOX1 and p62, but dramatically less potent in inducing the expression of other Nrf2 target genes such as aldo-ketoreductases.^33^ Notably, other phytocannabinoids, such as CBC and CBG, were found to be less potent in inducing HMOX1, and their acidic forms were completely inactive.

These observations provided a rationale to evaluate the phytocannabinoids isolated in this study for Nrf2 activation. Nrf2 transcriptional activity was analyzed in the HaCaT-ARE-Luc cell line. As a positive control for Nrf2 activation, the cells were treated with 20 μM of the antioxidant *tert*-butyl-hydroquinone (TBHQ), for which the effect was taken as 100%.

**Figure 5:**
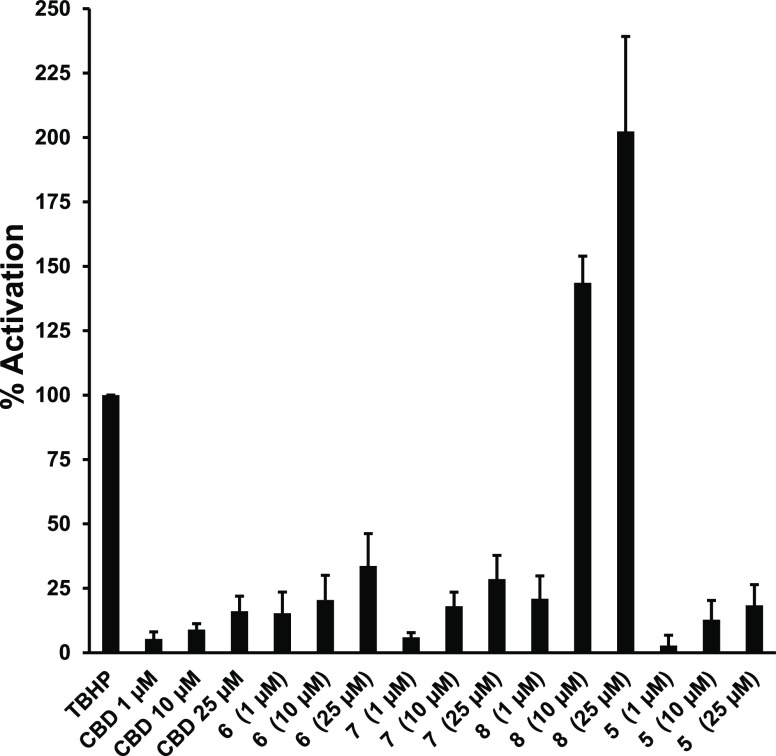
Effects of selected phytocannabinoids on Nrf2 activity. HaCaT-ARE-Luc cells (15 × 10^4^ cells/mL) were treated with 1–10–25 μM concentrations of each compound for 6 h. Luciferase activity was measured in the cell lysates, and the results are represented as percentage-fold induction relative to 20 μM *tert*-butyl-hydroquinone (TBHQ), taken as 100%.

**Figure 6:**
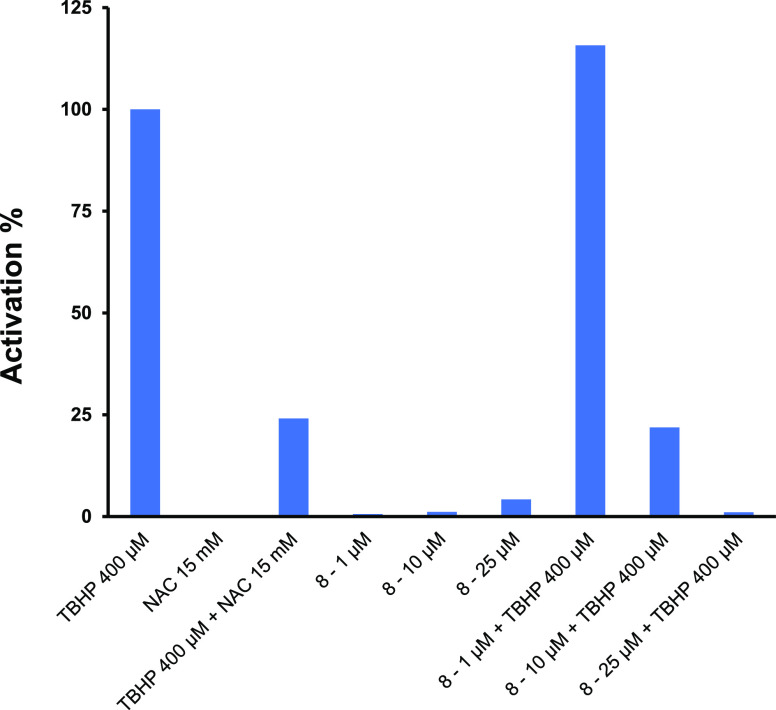
Intracellular accumulation of ROS detected using 2′,7′-dihydrofluorescein-diacetate (DCFH-DA) in HaCaT cells. *tert*-Butyl-hydroperoxide (TBHP) (0.4 mM) was used as a standard (100%) ROS producer, while *N*-acetylcysteine (NAC) (15 mM) was used as a positive control that inhibited TBHP-induced ROS production. Cells were treated with increasing concentrations (1–10–25 μM) of hexocannabitriol (**8**).

Apart from hexocannabitriol (**8**), which showed a very potent Nrf2 activation effect, with 20% induction at 1 μM, 144% at 10 μM, and 202% at 25 μM all the other test compounds, including CBD, proved to be very moderate Nrf2 activators (Figure 5). It was then evaluated if the potent activation of the Nrf2 pathway shown by hexocannabitriol was mediated by ROS induction. Figure 6 shows that **8** did not induce ROS production, although it was able to reduce the *tert*-butyl-hydroperoxide (TBHP)-induced ROS production in a concentration-dependent manner. Thus, at 25 μM, **8** completely abolished the ROS production induced by 400 μM TBHP. These results suggest that the potent activation of the Nrf2 pathway detected for hexocannabitriol is not an indirect effect mediated by ROS induction and may be the result of a direct stabilization of Nrf2 (sulphoraphane-like).^32^

Compounds tested in this study belong to the same structural series, presenting point variations located at the *p*-menthyl subunit, especially regarding the positions C-1 and C-2 that range from a double bond in CBD to the dihydroxylation in **6** and **7**, and a furan ring formation in **4** and **5**. The single structural arrangement triggering potent Nrf2 activation is the 2-hydroxy-Δ^1,7^ hexomethylene present in hexocannabitriol, while only modest activity is present both in the parent compound (CBD) and in other oxidized analogues. This very strict structure–activity requirement suggests the crucial role played by this molecular region in the interaction with the target. The direct Nrf2 activator sulphoraphane is a strongly electrophilic compound, and it is believed to act via modification of cysteine residues of the Kelch-like ECH-associated protein 1 (KEAP1).^34,35^ Conversely, given the nonelectrophilic nature of hexocannabitriol, a different mechanism should operate, which may be worth addressing in future studies.

The development of chemopreventive agents, able to enhance the transcription of Nrf2 target genes, and induction of cytoprotective enzymes hold significant promise for protection against a diversity of environmental stresses that contribute to the burden of inflammation, cancer, and other degenerative diseases. The present study, besides disclosing the structures of three unprecedented phytocannabinoids (**6**–**8**) that can be viewed as CBD counterparts of Δ^9^-THC metabolites, has also revealed that one of them (**8**) is a potent Nrf2 inducer. Although further research is needed to better establish the mechanism and potential value of the effects described in this study, hexocannabitriol (**8**) represents a significant addition in the rich repertoire of bioactivities ascribed to phytocannabinoids.

## Experimental Section

### General Experimental Procedures

Optical rotations (CH_3_OH) were measured at 589 nm on a P2000 (JASCO Europe s.r.l., Cremella, Italy) polarimeter. ^1^H (700 MHz) and ^13^C (175 MHz) NMR spectra were measured on a Bruker Avance 700 spectrometer (Bruker, Billerica, MA, USA). Chemical shifts are referenced to the residual solvent signal (CDCl_3_: δ_H_ 7.26, δ_C_ 77.0). Homonuclear ^1^H connectivities were determined by COSY (correlation spectroscopy) experiments. Through-space ^1^H connectivities were evidenced using a NOESY (nuclear Overhauser enhancement spectroscopy) experiment. One-bond heteronuclear ^1^H–^13^C connectivities were determined by the HSQC (heteronuclear single quantum correlation) experiment; two- and three-bond ^1^H–^13^C connectivities, by gradient-HMBC (heteronuclear multiple bond correlation) experiments optimized for a ^2,3^*J* of 8 Hz. HRESIMS experiments were performed on an LTQ-Orbitrap mass spectrometer equipped with an ESI interface and Excalibur data system. MPLC-DAD separations were performed on an Interchim instrument, puriFlash XS 520 Plus (Sepachrom s.r.l., Milan, Italy), using a Purezza-Daily C_18_ cartridge (60 Å 50 μm, Size 330 (475 g) and 4 (5.9 g)) and a Purezza-SpheraPlus cartridge (C_18_ 100 Å 25 μm, size 12). RP-HPLC–UV–vis separations were performed on an Agilent instrument, using a 1260 Quat Pump VL system, equipped with a 1260 VWD VL UV–vis detector, using Luna 10 and 5 μm C_18_ 100 Å 250 × 10 mm columns, a Luna 3 μm polar C_18_ 100 Å 150 × 3 mm column, and a Synergi 4 μm polar-RP 80 Å 250 × 4.60 mm column and a Rheodyne injector. Thin-layer chromatography (TLC) was performed on plates coated with silica gel 60 F254 (Merck, 0.25 mm). Chemicals and solvents were from Merck Life Science and were used without any further purification unless stated otherwise.

### Plant Material

*Cannabis sativa* L. specimens used were grown and authenticated at Indena SpA farms and generously provided for this study.

### Extraction and Isolation

*C. sativa* hemp threshing residues (1.0 kg, previously ground) were extracted in a percolator with 80% EtOH (v/v) at room temperature until exhaustion of the biomass. The leachates were filtered, collected, and concentrated to a small volume. The mixture was diluted with EtOH/water to 70% EtOH concentration and 15% dry residue. The pH was adjusted to 6.4, and the solution was quickly heated at 95 °C for phytocannabinoid decarboxylation. The decarboxylated mixture was concentrated and diluted with EtOH to afford a 30% EtOH solution. The suspension was extracted with *n*-hexane (4 × 300 mL). The polar organic phase was collected and concentrated to an oily matter, diluted in *n*-hexane/CH_2_Cl_2_ (8:2), and the suspension was filtered through an Arbocel (cellulose fibers) panel to clarify the solution. A first chromatographic purification step was carried out on silica gel eluting with *n*-hexane/CH_2_Cl_2_ (8:2). The CBD (**2**)-containing fractions were concentrated to a soft residue, and then CBD was crystallized from heptane and the supernatant was used for further investigation.

### LC-MS/MS and Molecular Networking

All LC-MS and LC-MS/MS experiments were performed on a Thermo LTQ-XL ion trap mass spectrometer (Thermo Fisher Scientific Spa, Rodano, Italy) coupled to a Thermo Ultimate 3000 HPLC system (Agilent Technology, Cernusco sul Naviglio, Italy). The LC-MS was carried out on a Kinetex 2.6 μm polar C_18_ 100 Å (100 × 3 mm) column (Phenomenex, Torrance, CA, USA), using 0.1% v/v of HCOOH in H_2_O (solvent A) and CH_3_CN (solvent B) as mobile phase. The gradient elution was optimized as follows: 50% B for 3 min, 50% to 95% B over 20 min, held 2 min, followed by a further 5 min of the initial conditions. The total run time, including the column wash and the equilibration, was 28 min, flow rate 0.5 mL/min, injection volume 5 μL. The MS and MS^*n*^ spectra, in the positive and in the negative modes, were recorded in Data Dependent Acquisition mode inducing fragmentation of the most intense five peaks for each scan. Source conditions: spray voltage 3.5 kV (positive mode) and 2.9 kV (negative mode); capillary voltage 25 V; source temperature 320 °C; normalized collision energy 25%. The acquisition range was *m*/*z* 150–1500. Although the spectra were recorded in both the positive and the negative modes, only the data obtained in positive mode have been taken into account.

A molecular network was created with the feature-based molecular networking (FBMN) workflow^36^ on GNPS (https://gnps.ucsd.edu). The mass spectrometry data first were processed with the software MZmine version 2.51,^37^ and the results were exported to GNPS for FBMN analysis. In detail, as initial data preprocessing, the mass detection was realized to use a centroid mass detector with the noise level set to 1.0 × 10^3^ for the MS1 level and the MS2 level at 1.0 × 10^2^. The chromatogram building was carried out utilizing ADAP (Automated Data Analysis Pipeline) chromatogram builder with a minimum group size of scans of 5, a minimum group intensity threshold of 5.0 × 10^2^, a minimum highest intensity of 1.0 × 10^3^, and a *m*/*z* tolerance of 0.008 *m*/*z* or 10 ppm. The chromatogram deconvolution by baseline cutoff was used with the following settings: min peak height 1000; peak duration range 0–10, and baseline level 0. Chromatograms were deisotoped using the isotopic peaks grouper algorithm with an *m*/*z* tolerance of 0.008 *m*/*z* or 10 ppm; retention time tolerance of 0.2 min; maximum charge of 1; representative isotope most intense. The data were filtered by removing all MS/MS fragment ions within ±17 Da of the precursor *m*/*z*. MS/MS spectra were window filtered by choosing only the top six fragment ions in the ±50 Da window throughout the spectrum. The precursor ion mass tolerance was set to 2 Da, and the MS/MS fragment ion tolerance to 0.5 Da. A molecular network was then created where edges were filtered to have a cosine score above 0.6 and more than six matched peaks. Further, edges between two nodes were kept in the network if and only if each of the nodes appeared in respective top 10 most similar nodes. Finally, the maximum size of a molecular family was set to 100, and the lowest scoring edges were removed from molecular families until the molecular family size was below this threshold. The spectra in the network were then searched against GNPS spectral libraries. The library spectra were filtered in the same manner as the input data. All matches kept between network spectra and library spectra were required to have a score above 0.7 and at least six matched peaks. The DEREPLICATOR was used to annotate MS/MS spectra.^38^ The molecular networks were visualized using Cytoscape_v3.7.2 software.^39^

### Isolation of Pure Compounds

A part of the mother liquor (5.2 g) was subjected to an MPLC-DAD chromatographic purification on a C_18_ 60 Å 50 μm cartridge, size 330 (475 g), column volume (CV) 430 mL. The mobile phase was a mixture of (A) water with a 0.1% formic acid, (B) acetonitrile, and (C) methanol with a gradient method as follows: starting conditions: 60% A–35% B–5% C for CV1; 50% A–45% B–5% C for CV2–4; 40% A–55% B–5% C for CV5–7; 30% A–65% B–5% C for CV8–10; 20% A–75% B–5% C for CV11–13; 10% A–85% B–5% C for CV14–16; 95% B–5% C for CV17–20; 5% B–95% C for CV21–28. The flow rate was 50.0 mL/min. The UV detection wavelength was set at 275 nm. This separation afforded 18 fractions (labeled A–T) and led to isolation of cannabidiol (**2**, 1.7 g, fractions H and I) in a pure form. Other fractions required subsequent HPLC purification. Fraction C (eluted with a mixture of 50% A–45% B–5% C) was rechromatographed by RP-18 HPLC-UV using an elution gradient from 60% A–35% B–5% C to 50% A – 45% B–5% C in 23 min, flow rate 0.5 mL/min, to yield compound **4b** (2.5 mg, *t*_R_ 19 min). Fraction D (eluted with a mixture of 40% A–55% B–5% C) was rechromatographed by RP-18 HPLC-UV using the following elution gradient: 0–5 min = 50% A–50% B isocratic; 15–30 min = 45% A–55% B (flow rate 10.0 mL/min), affording **7** (2.0 mg, *t*_R_ 20.9 min) and **6** (2.2 mg, *t*_R_ 22.7 min) in pure state. Fraction E (eluted with a gradient from 40% A–55% B–5% C to 30% A–65% B–5% C) was separated by RP-18 HPLC-UV using the following elution gradient: from 50% A–50% B to 40% A–60% B in 15 min and then isocratic for 5 min. The flow rate was 0.5 mL/min, affording **8** (2.1 mg, *t*_R_ 11 min) in pure form. Fraction G (eluted with a mixture of 30% A–65% B–5% C) was separated by RP-18 HPLC-UV using an isocratic eluent of 45% A–50% B–5% C, flow rate 0.5 mL/min, to yield **5** (1.9 mg, *t*_R_ 21.0 min) and **4a** (18.5 mg, *t*_R_ 23 min). Fraction H (eluted with a mixture of 20% A–75% B–5% C) was separated by RP-MPLC using the following elution gradient: 0–2 CV (20 mL) = 40% A–55% B–5% C; 7–17 CV = 30% A–65% B–5% C; 17–22 CV = 20% A–75% B–5% C; 26–30 CV = 10% B–90% C. The flow rate was 15.0 mL/min, affording CBDA (4.6 mg, CV 9). Fraction R (eluted with a mixture of 5% B–95% C) was first separated by RP-MPLC using the following elution gradient: 0–10 CV (20 mL) = 5% A–90% B–5% C; 20–35 CV = 90% B–10% C; 36–41 CV = 5% B–95% C, flow rate 15.0 mL/min, and then fraction R4 (CV8) was further purified by RP-HPLC-UV using an elution gradient from 30% A–70% C to 100% C in 25 min, flow rate 0.5 mL/min, to yield cannabitwinol (**3**, 1.0 mg, *t*_R_ 21 min).

#### Compound **6**:

pale yellow amorphous solid; [α]_D_ +10.9 (0.2, MeOH); ^1^H NMR (CDCl_3_, 700 MHz) and ^13^C NMR (CDCl_3_, 175 MHz), Tables 2 and 3; HRESIMS *m*/*z* 349.2378 [M + H]^+^ (C_21_H_33_O_4_ requires *m*/*z* 349.2373).

#### Compound **7**:

pale yellow amorphous solid; [α]_D_ +12.4 (0.14, MeOH); ^1^H NMR (CDCl_3_, 700 MHz) and ^13^C NMR (CDCl_3_, 175 MHz), Tables 2 and 3; HRESIMS *m*/*z* 347.2220 [M + H]^+^ (C_21_H_31_O_4_ requires *m*/*z* 347.2217).

#### Hexocannabitriol (**8**):

pale yellow amorphous solid; [α]_D_ +19.2 (0.2, MeOH); ^1^H NMR (CDCl_3_, 700 MHz) and ^13^C NMR (CDCl_3_, 175 MHz), Tables 2 and 3; HRESIMS *m*/*z* 331.2276 [M + H]^+^ (C_21_H_31_O_3_ requires *m*/*z* 331.2273).

### Biological Testing

#### Nrf2 Activity Assays

HaCaT-ARE-Luc cells were cultivated in 96-well plates with 2 × 10^4^ cells/well in a CO_2_ incubator at 37 °C. For induction of Nrf2 activation the cells were treated with increasing concentrations of the test substances for 6 h. As a positive control, the cells were treated with 0.4 mM of the prooxidant TBHP. Luciferase activity was measured using a TriStar2 Berthold/LB942 multimode reader (Berthold Technologies) following the instructions of the luciferase assay kit (Promega, Madison, WI, USA). The background obtained with the lysis buffer was subtracted from each experimental value, and the percentages of induction were determined relative to TBHP (100% activation). The results represent the means of three independent experiments.

#### Intracellular Accumulation of ROS

ROS accumulation was detected using 2′,7′-dihydrofluorescein-diacetate (DCFH-DA). HaCaT cells (15  ×  10^3^ cells/well) were cultured in a 96-well plate in DMEM supplemented with 10% fetal bovine serum until the cells reached 80% confluence. For inhibition, the cells were pretreated with **7** for 30 min and then treated with 0.4 mM TBHP. Three hours later, the cells were incubated with 10 μM DCFH-DA in the culture medium at 37 °C for 30 min. Then, the cells were washed with PBS at 37 °C, and the production of intracellular ROS, measured by DCF fluorescence, was detected using the Incucyte FLR software. The data were analyzed by the total green object integrated intensity (GCU × μm^2^ × well) of the imaging system IncuCyte HD (Sartorius, Göttingen, Germany). *N*-Acetylcysteine (15 mM) was used as a positive control that inhibited TBHP-induced ROS production.

## Supporting Information Available

Selected MS/MS spectra; 1D and 2D NMR spectra for **6**–**8** (PDF)
