# Supplementary material for: A Nrf-2 Stimulatory Hydroxylated Cannabidiol Derivative from Hemp (Cannabis sativa)
Source: J Nat Prod. 2022 Mar 22;85(4):1089–97. doi: 10.1021/acs.jnatprod.1c01198 (PMC9040056; doi:10.1021/acs.jnatprod.1c01198)
Supplement: Supplementary file 1 — np1c01198_si_001.pdf [file np1c01198_si_001.pdf]

SUPPORTING INFORMATION

A Nrf-2 Stimulating Hydroxylated Cannabidiol Derivative from Hemp (*Cannabis sativa*)

Giuseppina Chianese,[†] Carmina Sirignano,[†] Emanuele Benetti,[‡] Vittoria Marzaroli [‡] Juan A. Collado,[#,§] Lauren de la Vega,[^] Giovanni Appendino,[‖] Eduardo Munoz,[#,§] and Orazio Taglialatela-Scafati[†,*]

[†]Department of Pharmacy, School of Medicine and Surgery, University of Naples Federico II, Via D. Montesano 49, 80131 Napoli, Italy

[‡] Indena SpA, via Don Minzoni, 6 - 20049 Settala, Milan, Italy

[#]Instituto Maimónides de Investigación Biomédica de Córdoba (IMIBIC), Córdoba, Spain.

[§]Departamento de Biología Celular, Fisiología e Inmunología, Universidad de Córdoba, Spain and Hospital Universitario Reina Sofía, Córdoba, Spain.

[^]Jacqui Wood Cancer Centre, Division of Cellular Medicine, School of Medicine, University of Dundee, UK.

[‖]Dipartimento di Scienze del Farmaco, Università del Piemonte Orientale, Largo Donegani 2, 28100, Novara, Italy

Figure S1. ^{1}H NMR spectrum of compound **6** (700 MHz) in $CDCl_3$

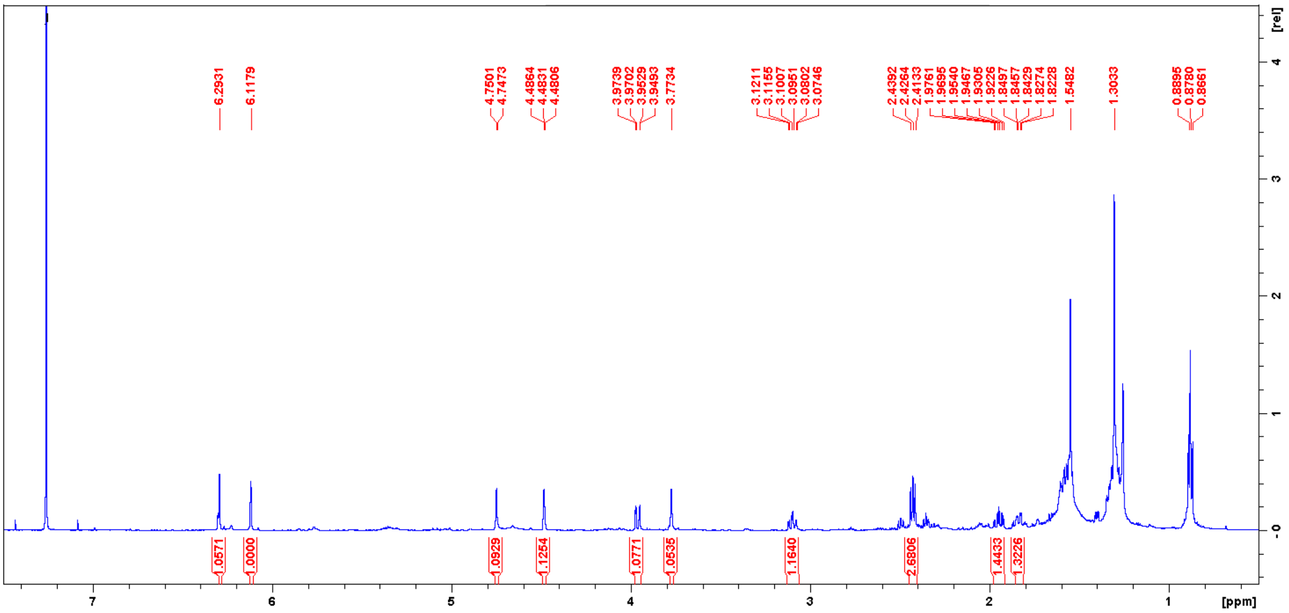

Figure S2. COSY 2D NMR spectrum of compound **6** (700 MHz) in $CDCl_3$

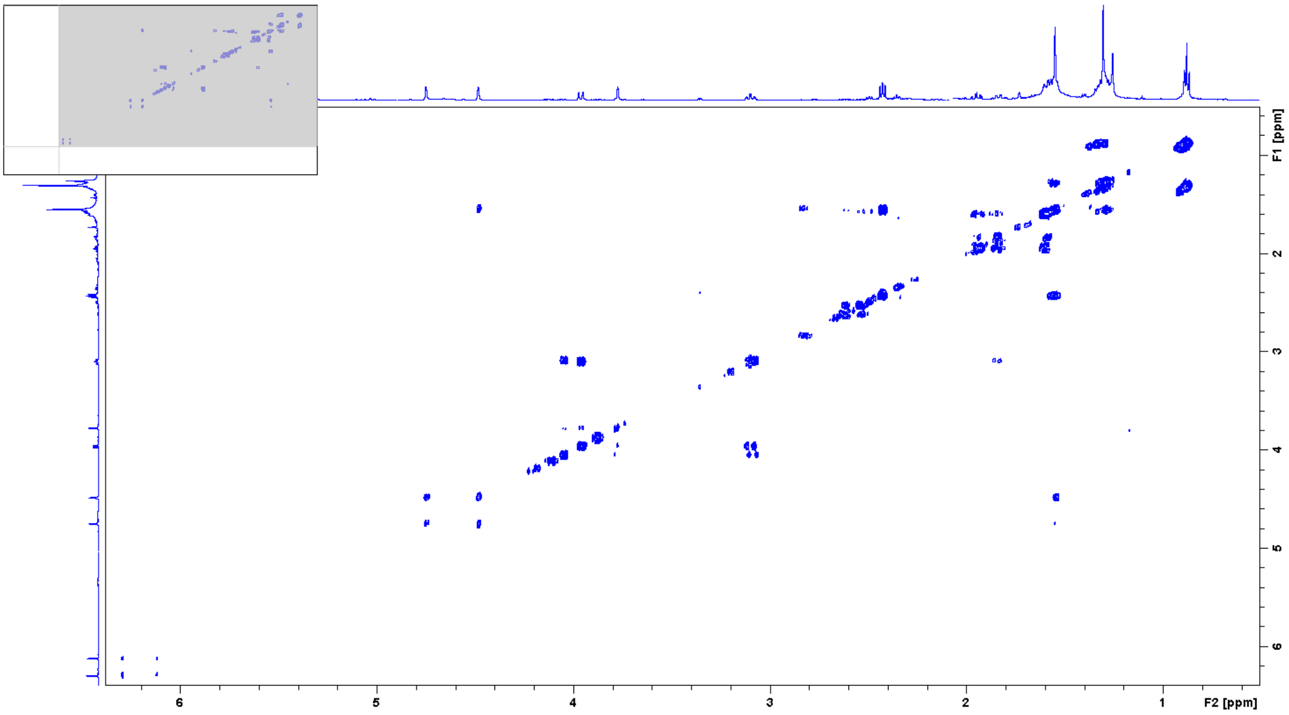

Figure S3. HMBC 2DNMR spectrum of compound **6** (700 MHz) in $CDCl_3$

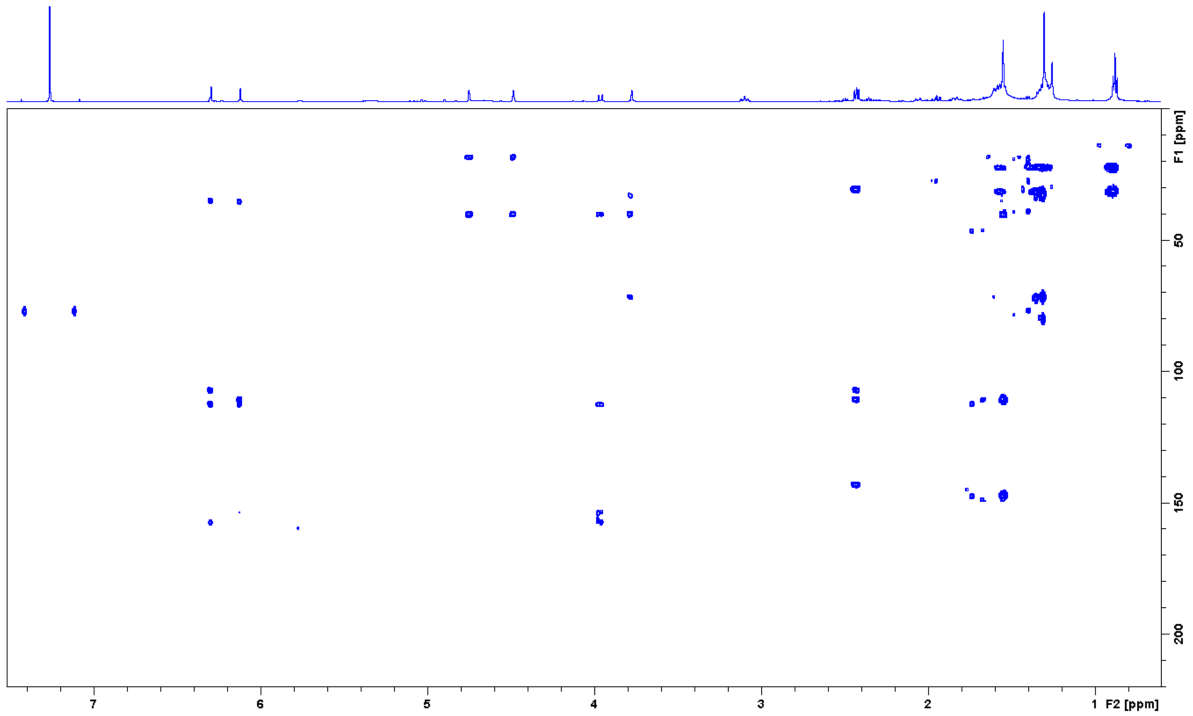

Figure S4. HSQC 2DNMR spectrum of compound **6** (700 MHz) in $CDCl_3$

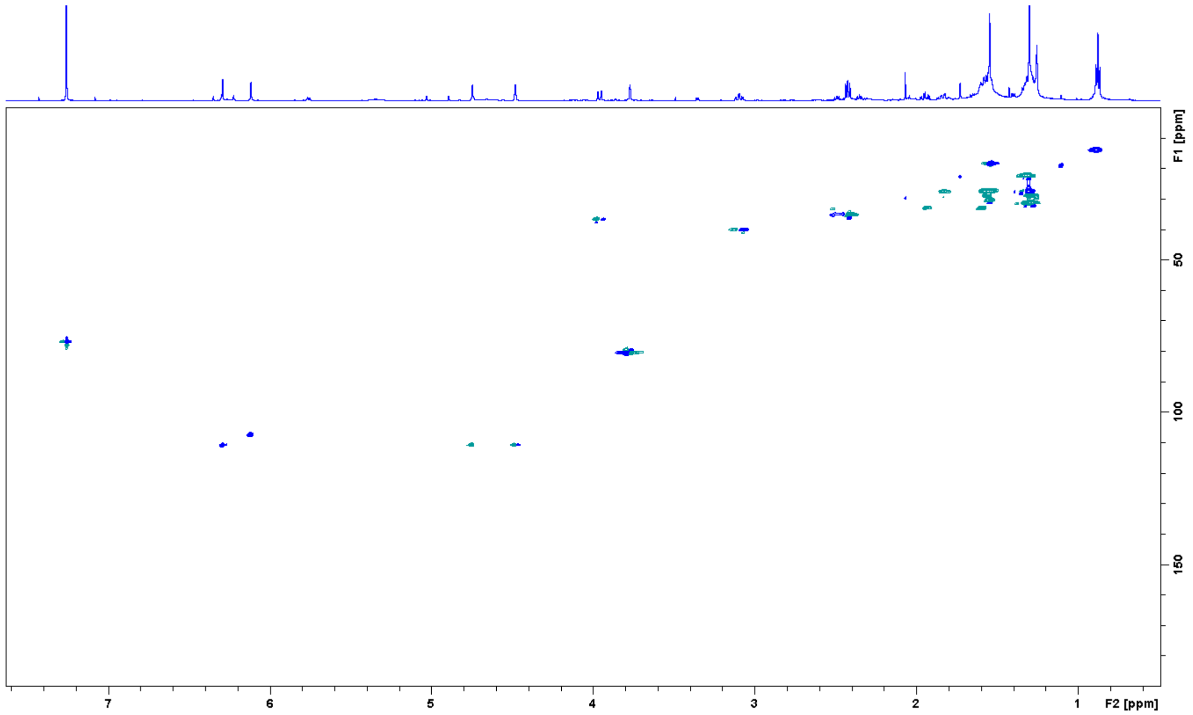

Figure S5. NOESY 2DNMR spectrum of compound **6** (700 MHz) in $CDCl_3$

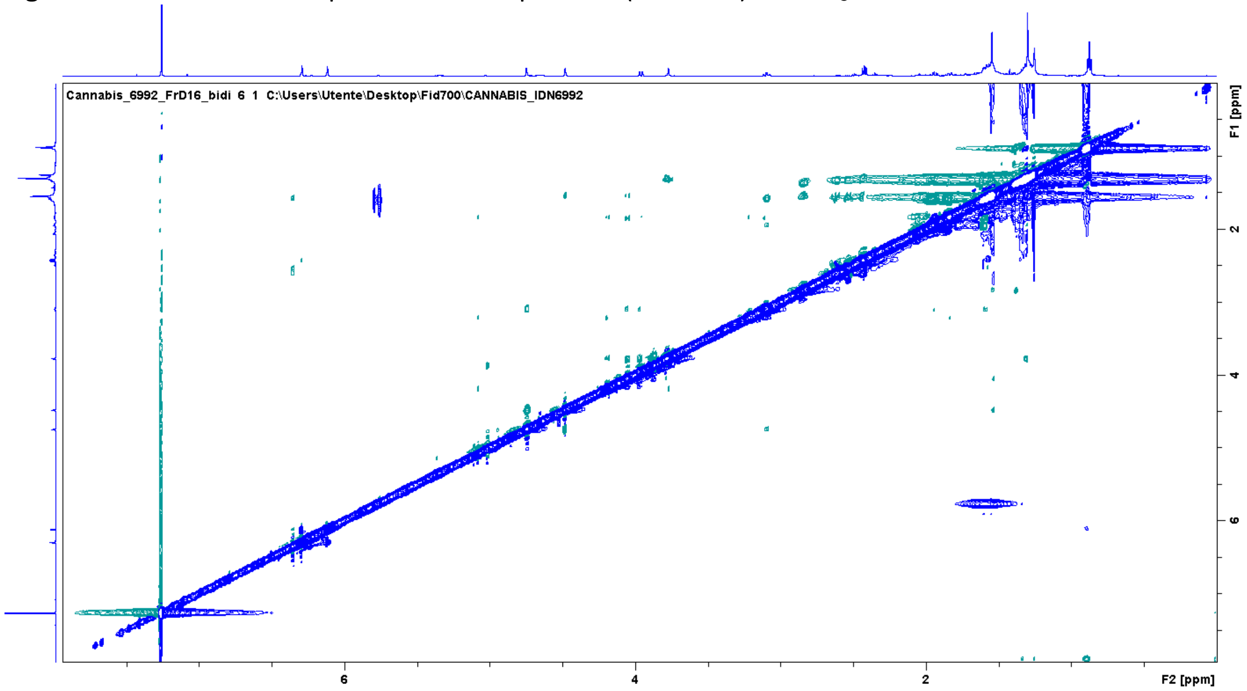

Figure S6. ^{1}H NMR spectrum of compound **7** (700 MHz) in $CDCl_3$

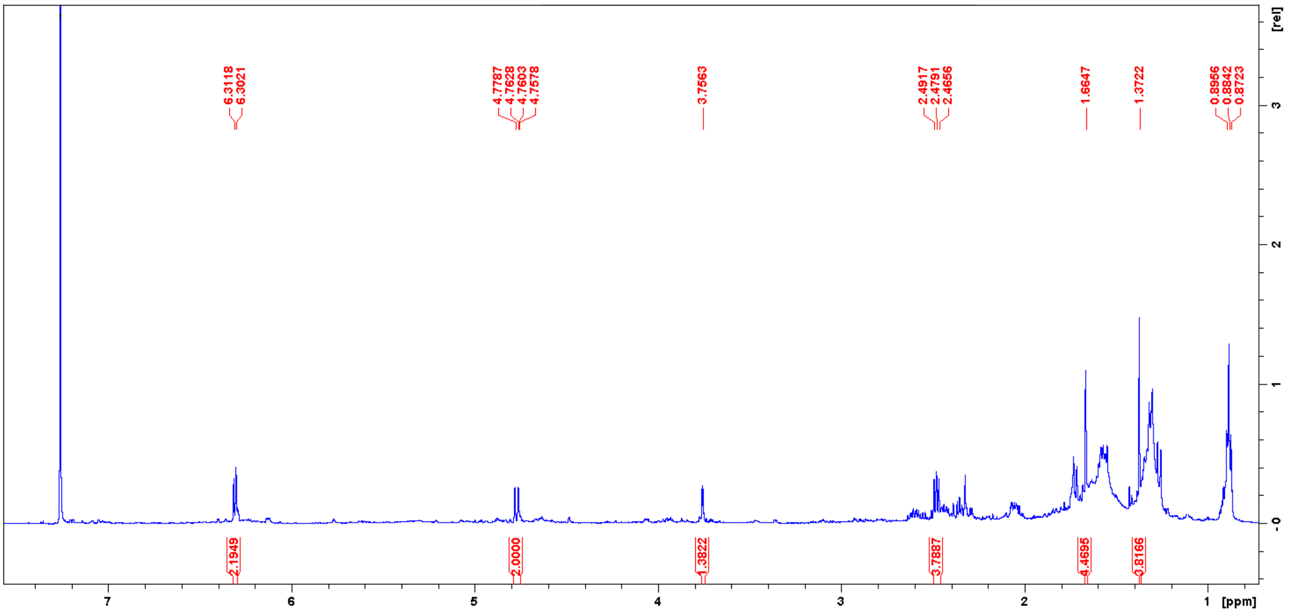

Figure S7. COSY 2D NMR spectrum of compound **7** (700 MHz) in $CDCl_3$

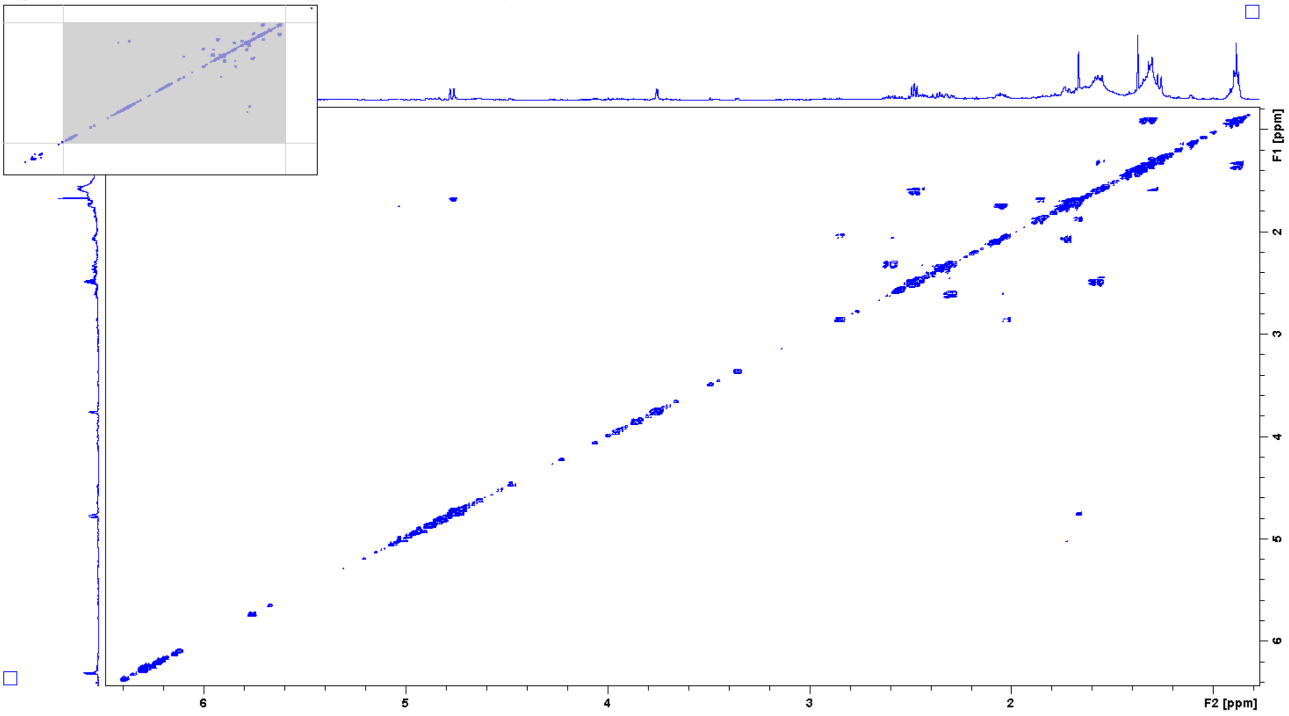

Figure S8. HMBC 2D NMR spectrum of compound **7** (700 MHz) in $CDCl_3$

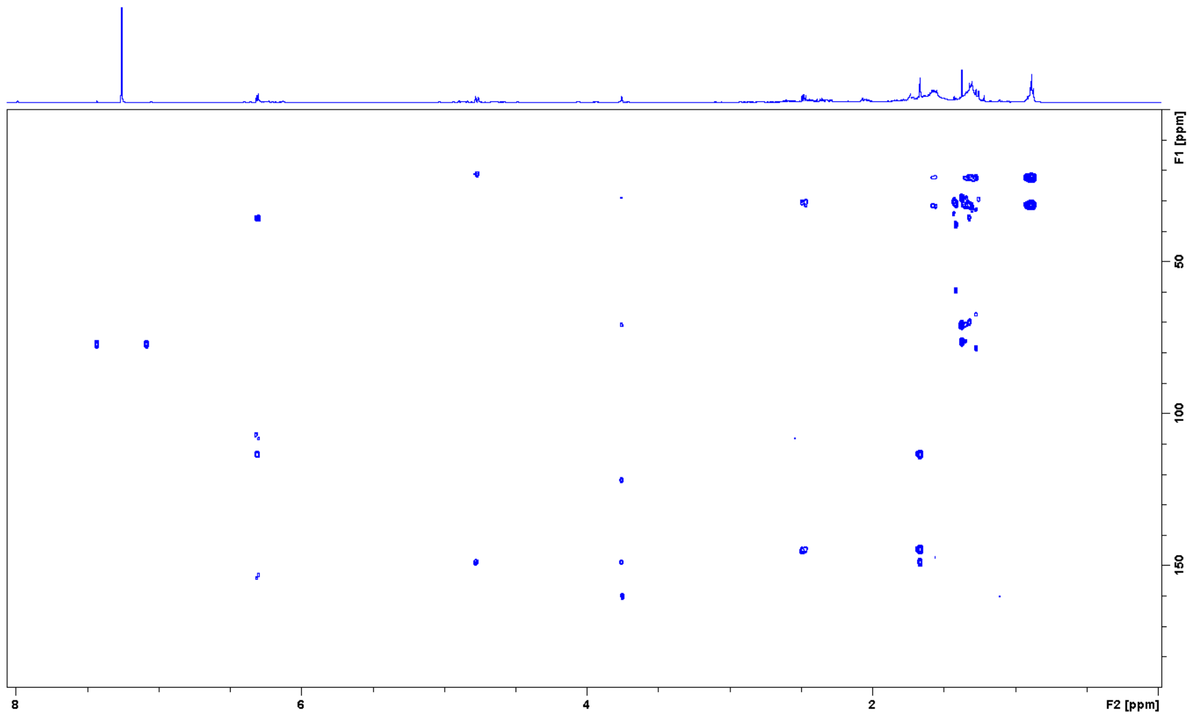

Figure S9. HSQC 2D NMR spectrum of compound **7** (700 MHz) in $CDCl_3$

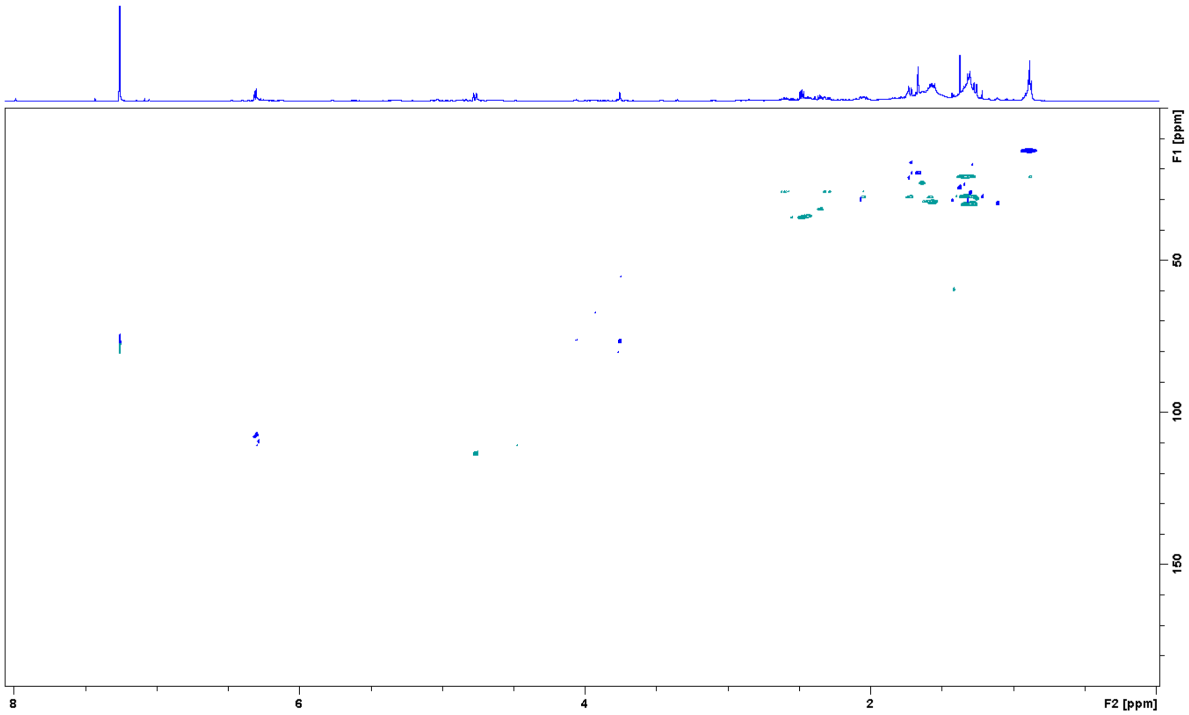

Figure S10. NOESY 2D NMR spectrum of compound **7** (700 MHz) in $CDCl_3$

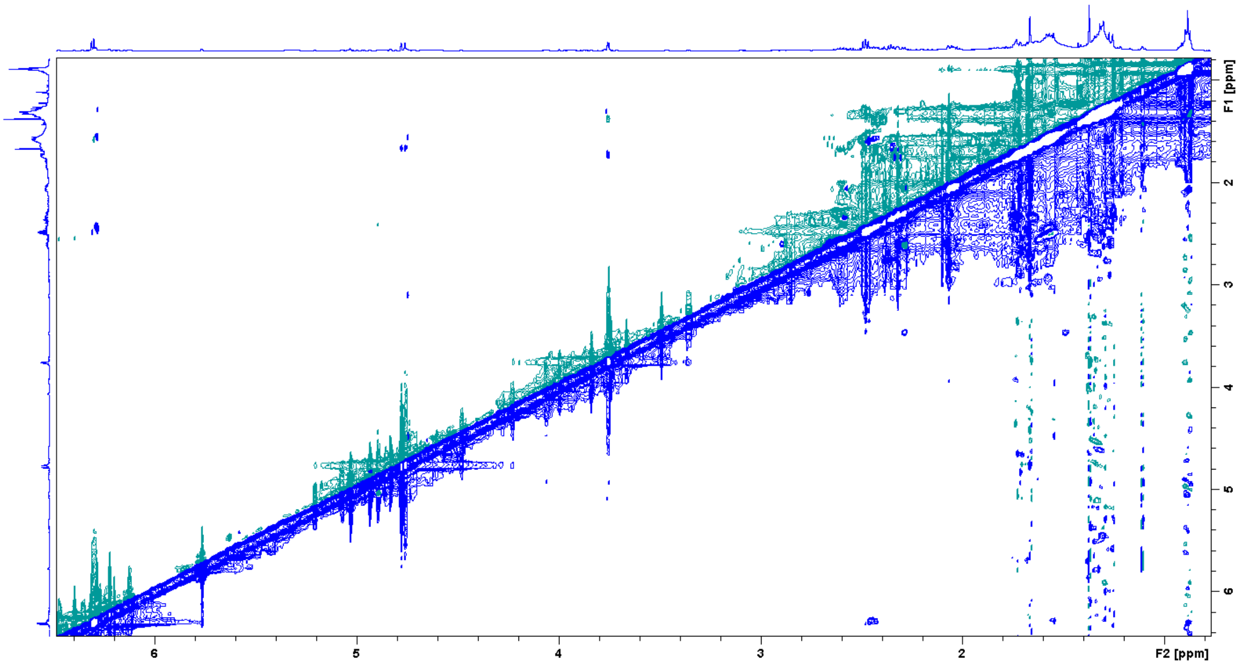

Figure S11. ^{1}H NMR spectrum of compound **8** (700 MHz) in $CDCl_3$

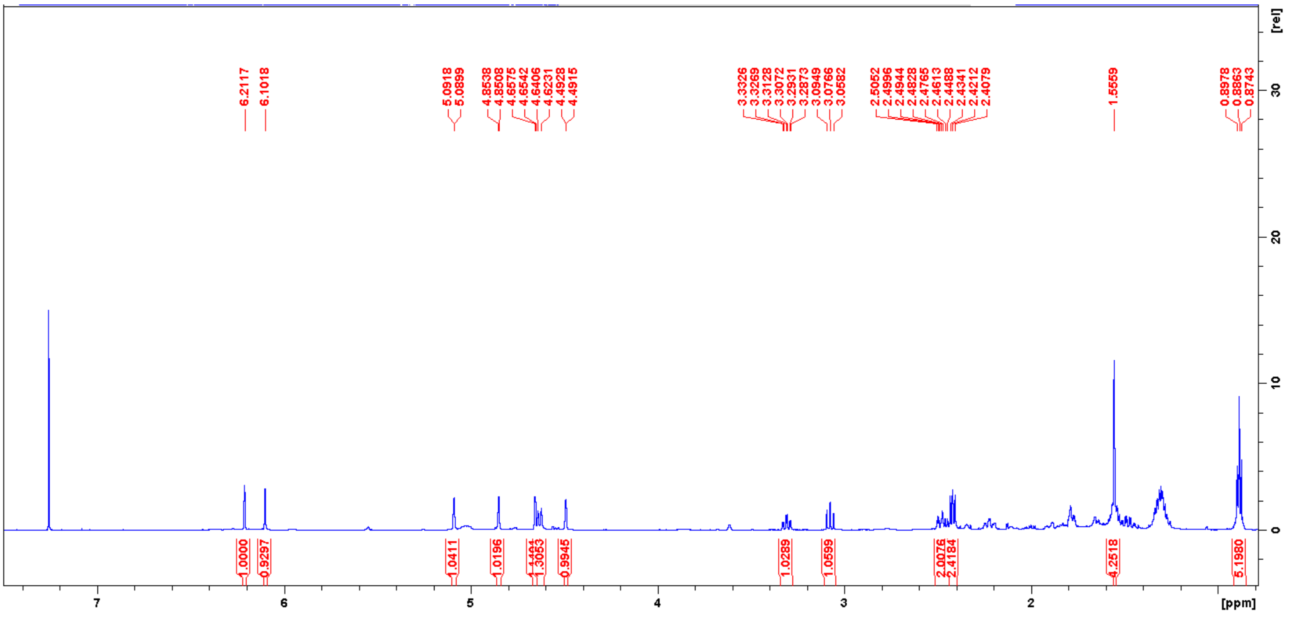

Figure S12. COSY 2D NMR spectrum of compound **8** (700 MHz) in $CDCl_3$

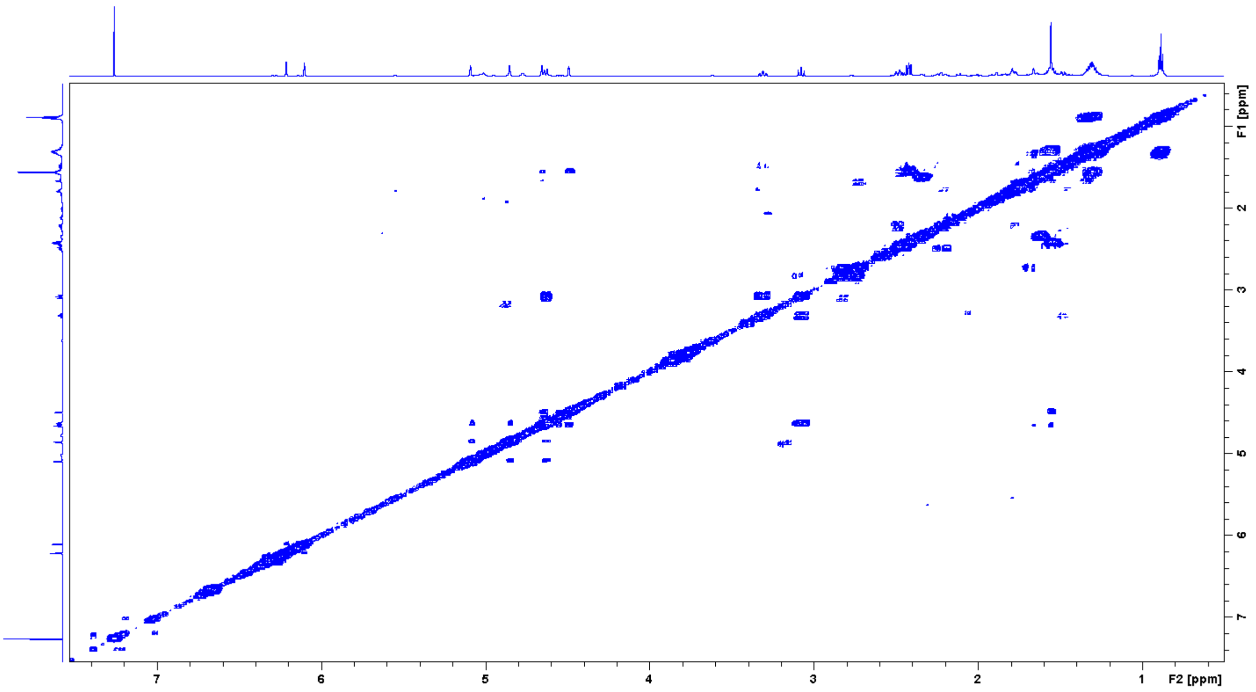

Figure S13. HMBC 2D NMR spectrum of compound **8** (700 MHz) in $CDCl_3$

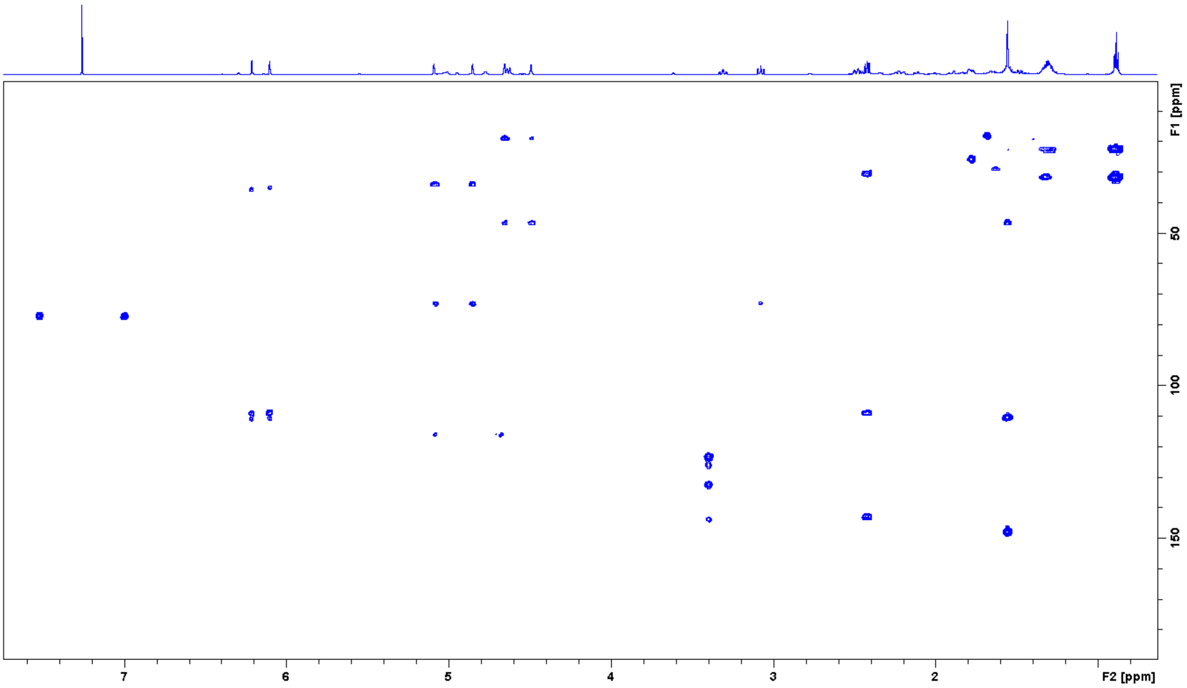

Figure S14. HSQC 2D NMR spectrum of compound **8** (700 MHz) in $CDCl_3$

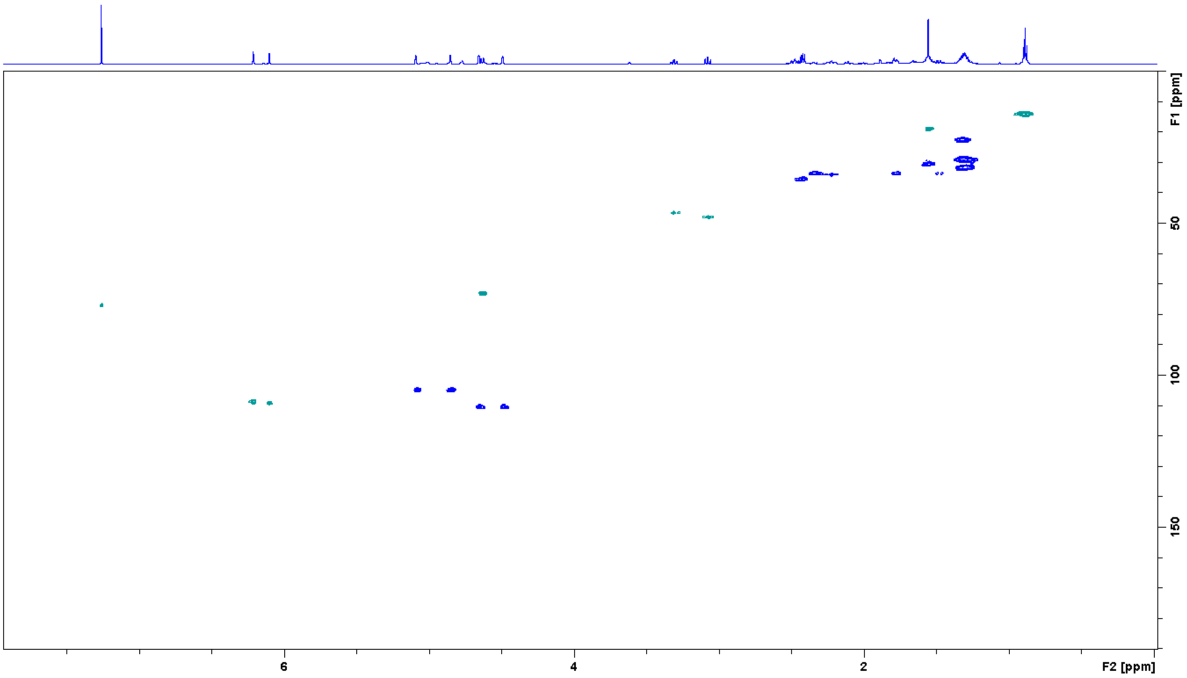

Figure S15. LC-MS chromatogram of the *Cannabis* supernatant extract fraction with: MS (on the right side) and MS/MS (on the left side) spectra of the nineteen annotate compounds reported in the Table 1.

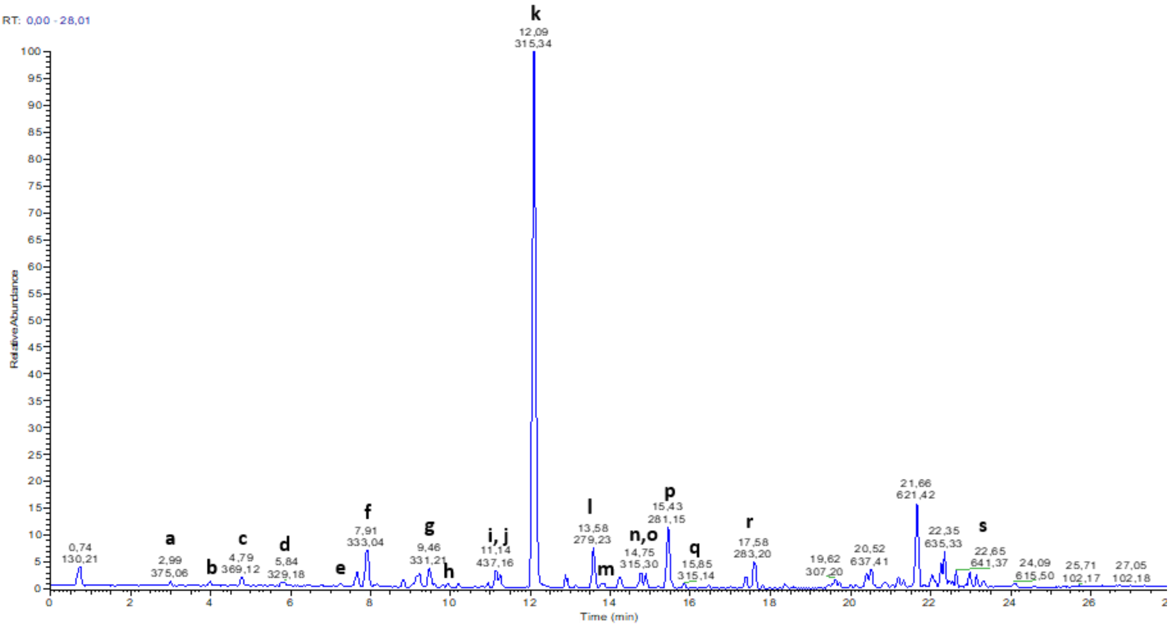

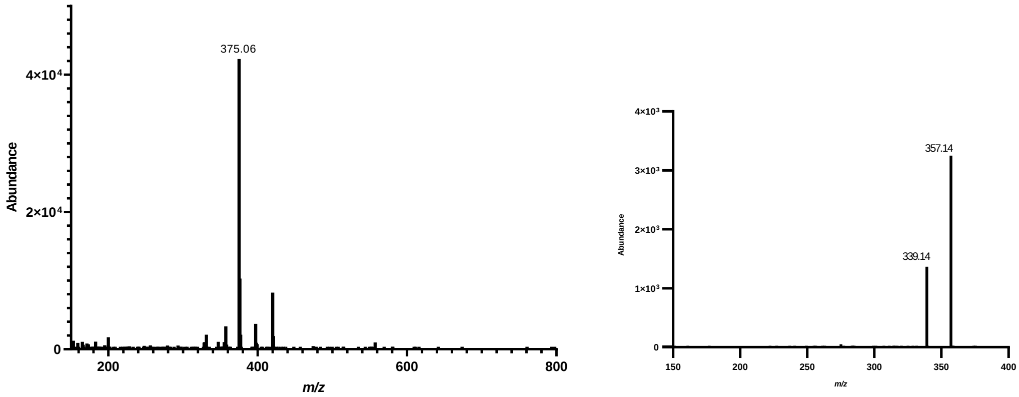

a.

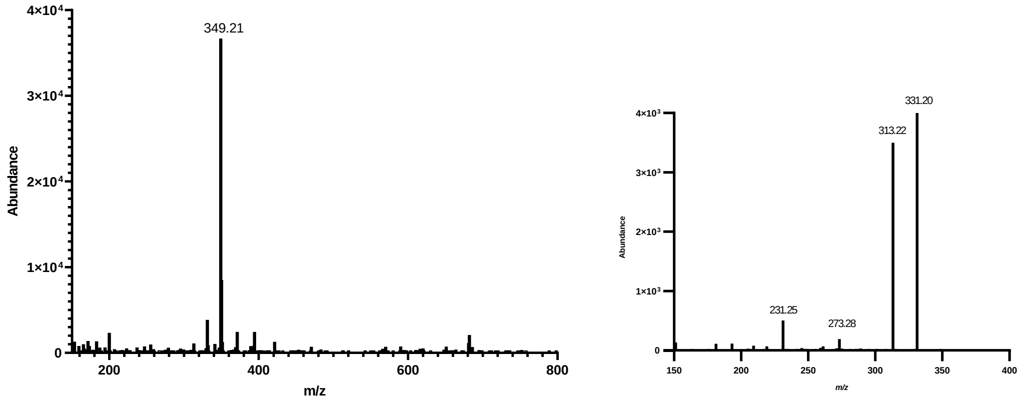

b.

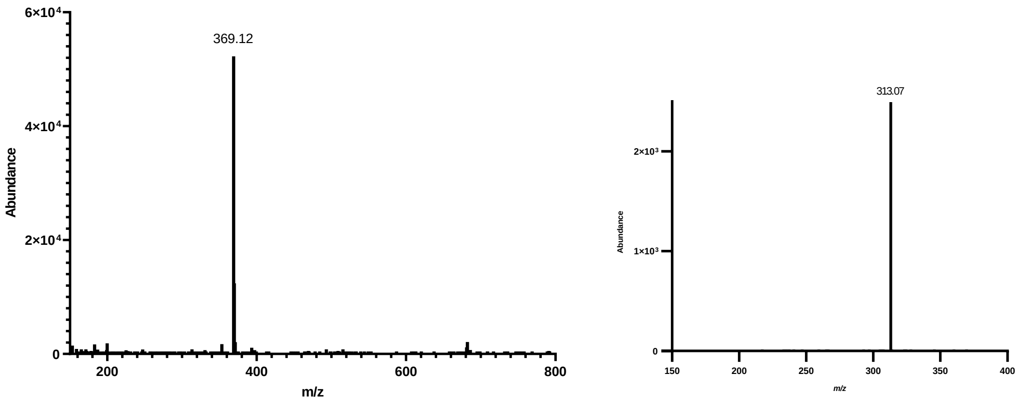
369.12
Abundance
m/z
313.07
Abundance
m/z

c.

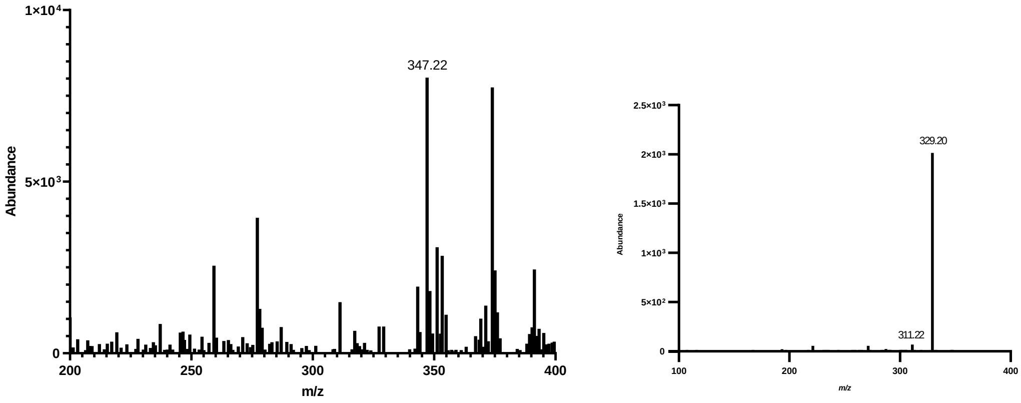
347.22
Abundance
m/z
329.20
311.22
Abundance
m/z

d.

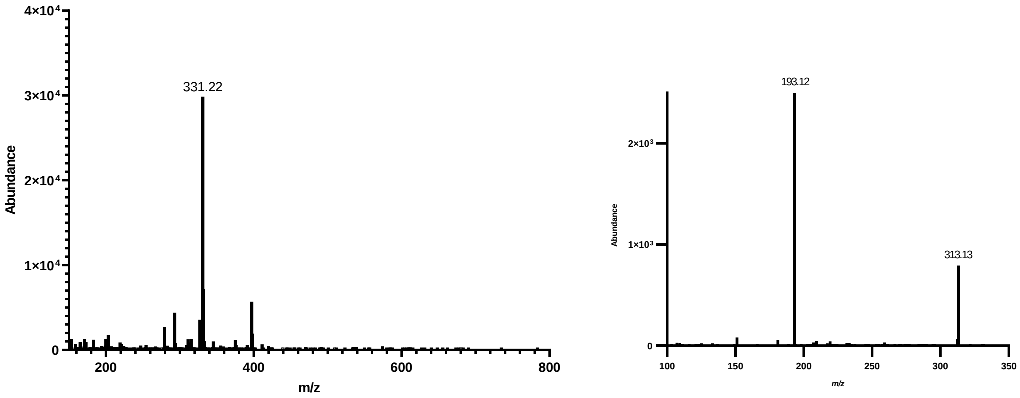
331.22
Abundance
m/z
193.12
313.13
Abundance
m/z

e.

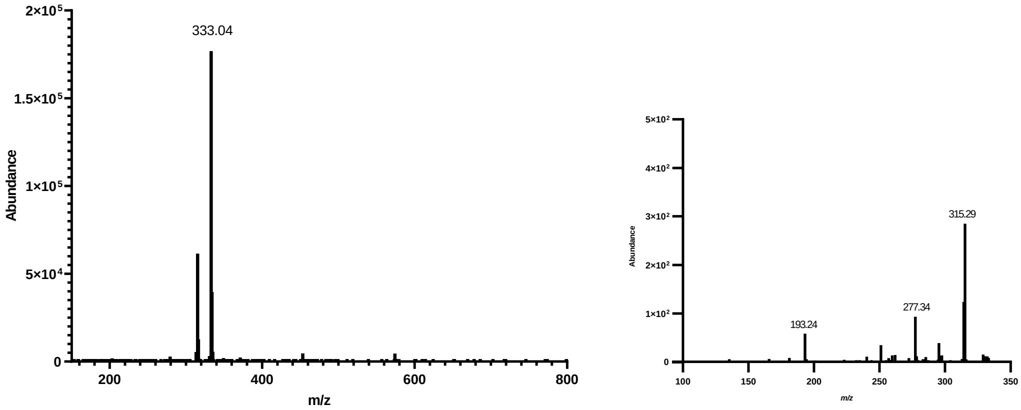
333.04
Abundance
m/z
315.29
277.34
193.24
Abundance
m/z

f.

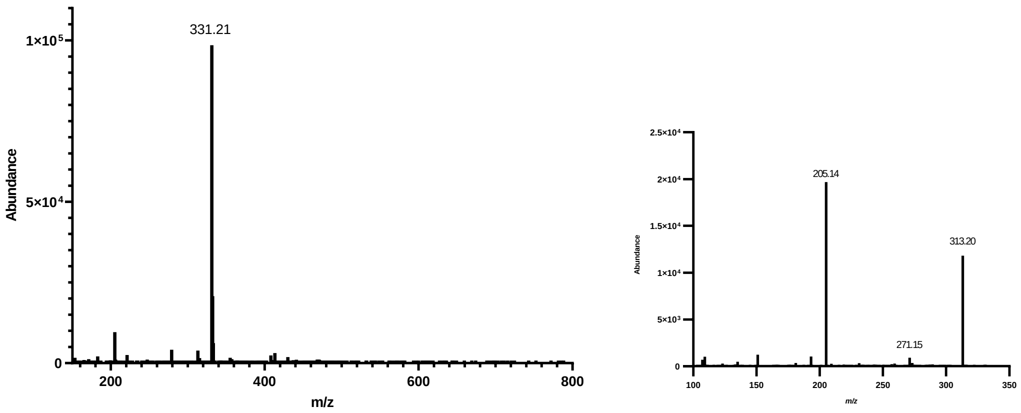
331.21
Abundance
m/z
205.14
313.20
271.15
Abundance
m/z

g.

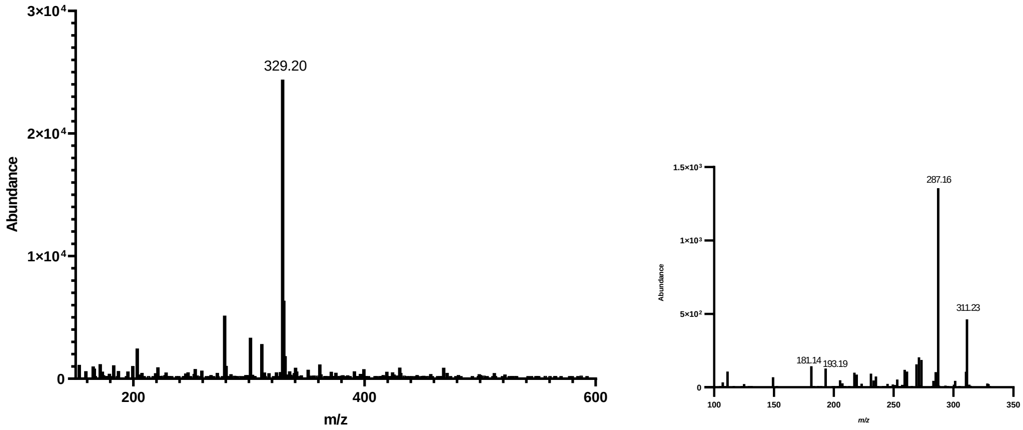
329.20
Abundance
m/z
287.16
311.23
181.14
193.19
Abundance
m/z

h.

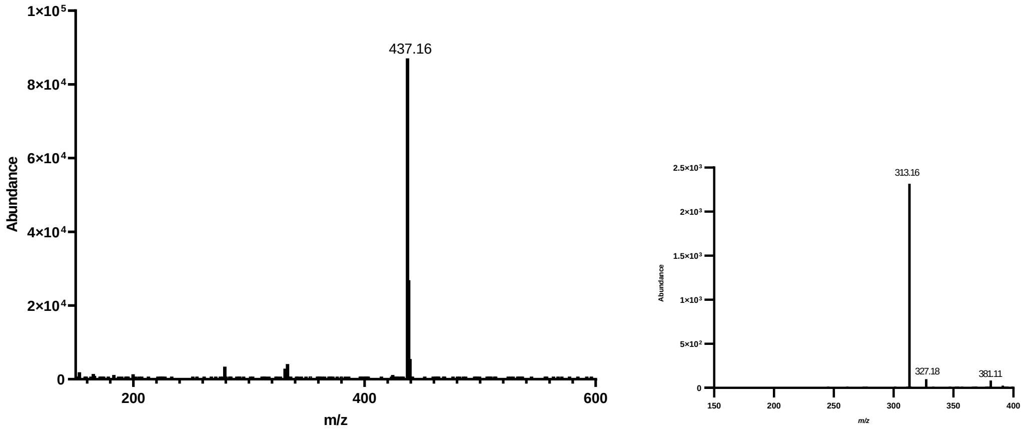
437.16
Abundance
m/z
313.16
327.18
381.11
Abundance
m/z

i.

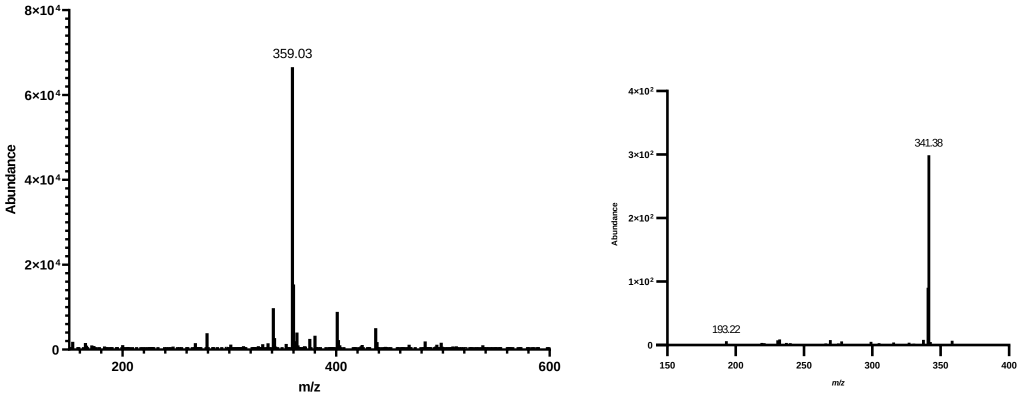
359.03
Abundance
m/z
341.38
193.22
Abundance
m/z

j.

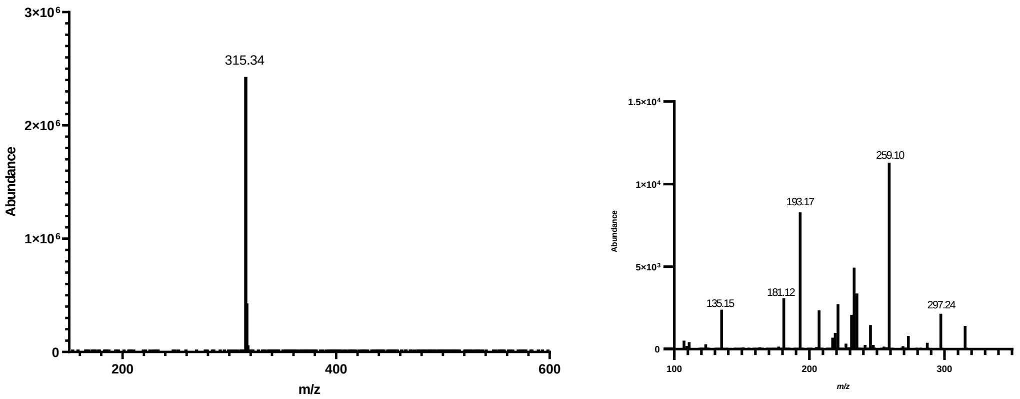
315.34
Abundance
m/z
259.10
193.17
135.15
181.12
297.24
Abundance
m/z

k.

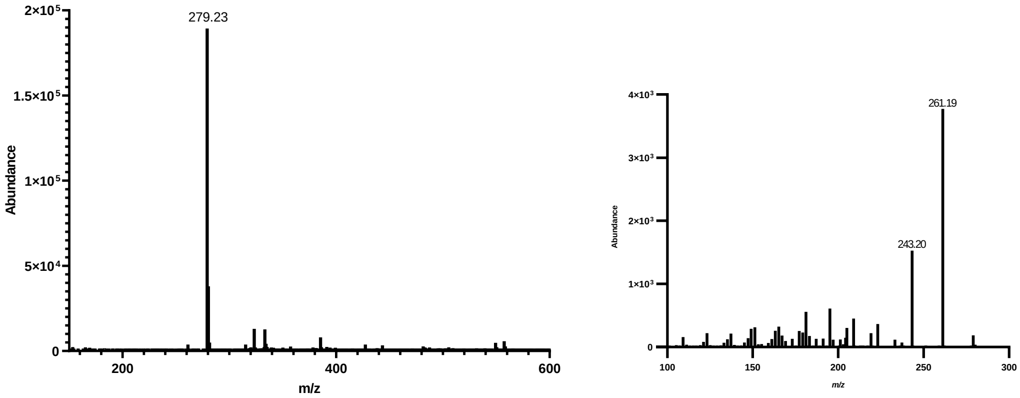
279.23
Abundance
m/z
261.19
243.20
Abundance
m/z

l.

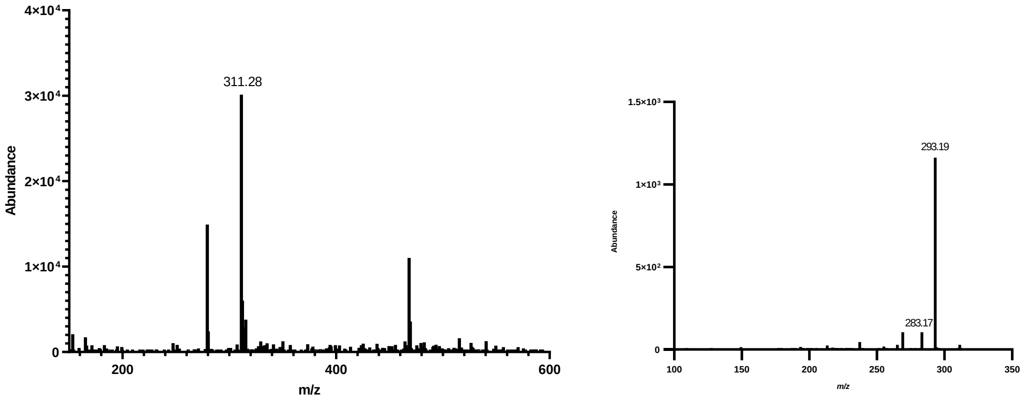
311.28
Abundance
m/z
293.19
283.17
Abundance
m/z

m.

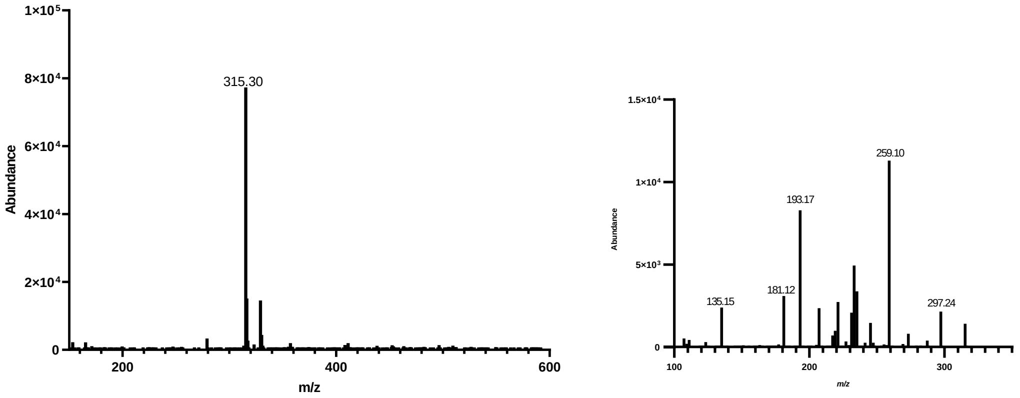
315.30
Abundance
m/z
259.10
193.17
135.15
181.12
297.24
Abundance
m/z

n.

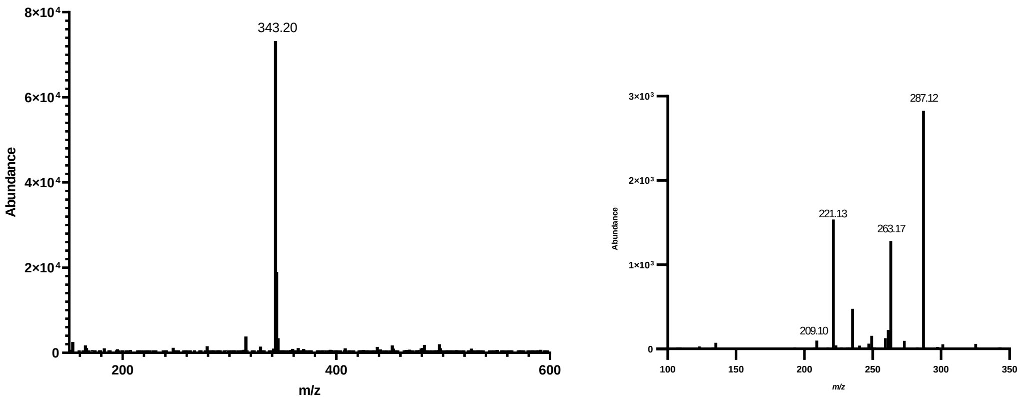
343.20
Abundance
m/z
287.12
221.13
263.17
209.10

o.

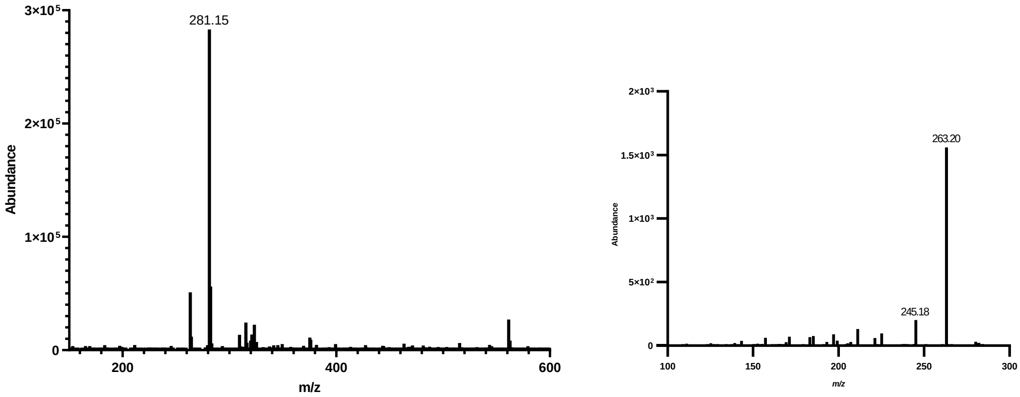
281.15
Abundance
m/z
263.20
245.18

p.

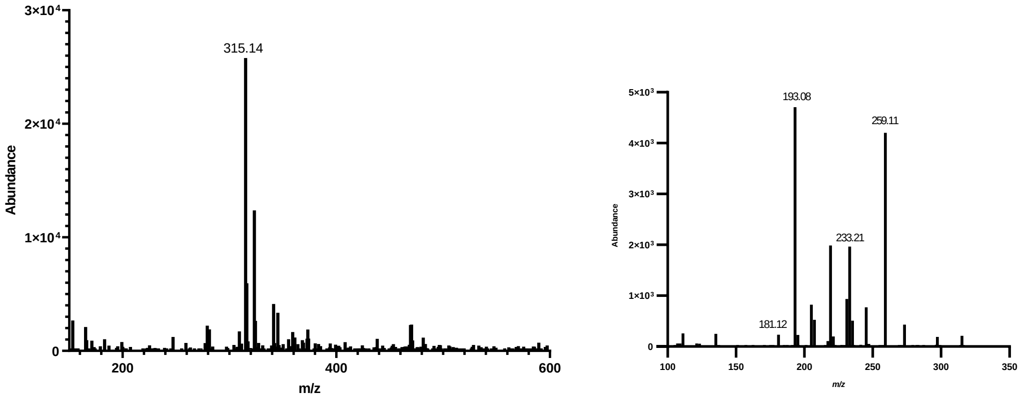
315.14
Abundance
m/z
193.08
259.11
233.21
181.12

q.

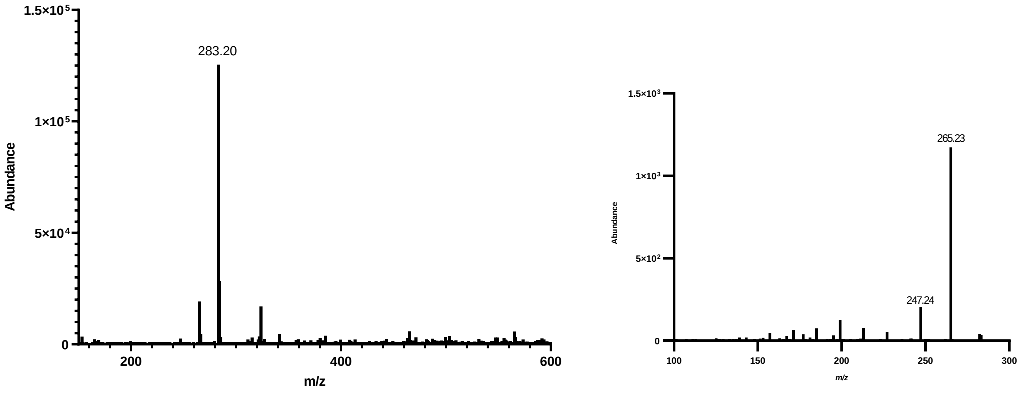
283.20
Abundance
m/z
265.23
247.24

r.

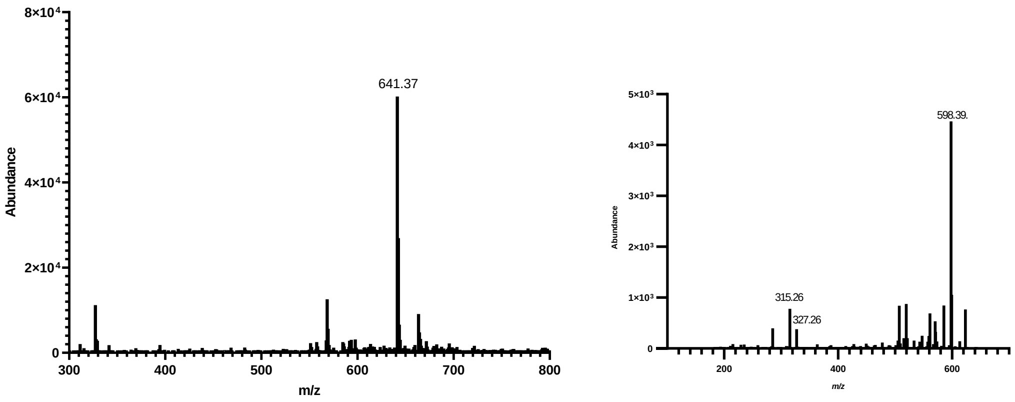

S.
